# A Minimalistic Coumarin Turn-On Probe for Selective Recognition of Parallel G-Quadruplex DNA Structures

**DOI:** 10.1021/acschembio.1c00134

**Published:** 2021-07-30

**Authors:** Marco Deiana, Ikenna Obi, Måns Andreasson, Shanmugam Tamilselvi, Karam Chand, Erik Chorell, Nasim Sabouri

**Affiliations:** † Department of Medical Biochemistry and Biophysics, Umeå University, 90187 Umeå, Sweden; ‡ Department of Chemistry, Umeå University, 90187 Umeå, Sweden

## Abstract

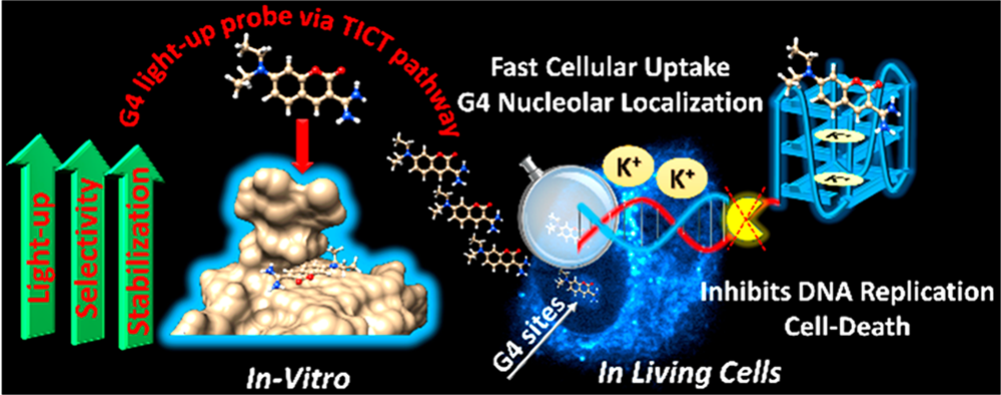

G-quadruplex (G4) DNA structures are widespread in the human genome and are implicated in biologically important processes such as telomere maintenance, gene regulation, and DNA replication. Guanine-rich sequences with potential to form G4 structures are prevalent in the promoter regions of oncogenes, and G4 sites are now considered as attractive targets for anticancer therapies. However, there are very few reports of small “druglike” optical G4 reporters that are easily accessible through one-step synthesis and that are capable of discriminating between different G4 topologies. Here, we present a small water-soluble light-up fluorescent probe that features a minimalistic amidinocoumarin-based molecular scaffold that selectively targets parallel G4 structures over antiparallel and non-G4 structures. We showed that this biocompatible ligand is able to selectively stabilize the G4 template resulting in slower DNA synthesis. By tracking individual DNA molecules, we demonstrated that the G4-stabilizing ligand perturbs DNA replication in cancer cells, resulting in decreased cell viability. Moreover, the fast-cellular entry of the probe enabled detection of nucleolar G4 structures in living cells. Finally, insights gained from the structure–activity relationships of the probe suggest the basis for the recognition of parallel G4s, opening up new avenues for the design of new biocompatible G4-specific small molecules for G4-driven theranostic applications.

## Introduction

G-quadruplexes (G4s) are four-stranded non-B DNA helical structures formed by the stacking of four in-plane guanine bases (G-quartets) stabilized through Hoogsteen-type hydrogen bonding and coordinated by a central metal ion (usually K^+^ or Na^+^).^1−4^ Extensive and detailed biophysical and structural studies have highlighted an impressive diversity of G4 topologies (including parallel, antiparallel, and hybrid structures) depending on the number of G-quartets, the strand orientation, the loop composition, and the nature of the stabilizing cation.^5,6^ Compelling evidence clearly implicates G4 motifs in key biological processes, including telomere maintenance,^7,8^ translational^9−11^ and transcriptional regulation,^12,13^ and DNA replication.^14,15^ Recent studies using G4-specific monoclonal antibodies^16−18^ or optical reporters^19−23^ have provided evidence for G4 formation in cells. Furthermore, bioinformatics^24,25^ and sequencing^26,27^ approaches have highlighted the widespread distribution of potential G4-forming sequences in the human genome. G4 motifs are enriched in the promoter regions of oncogenes, at telomeres, and in the untranslated regions (UTRs) of mRNAs.^5^ Indeed, these sites are now viewed as attractive targets for small molecule therapeutics and diagnostic agents.^28,29^ For instance, by targeting G4s with small molecules it is possible to control the expression/transcription of otherwise undruggable oncogenic proteins such as c-*MYC*^30−32^ and *KRAS*.^33^

A longstanding goal in the G4 field has been to develop topology-specific G4-interactive compounds capable of detecting intracellular G4s located in the nucleus. Different classes of optical probes have been reported to fluoresce upon G4 binding,^29,34^ but the overwhelming majority of the designed ligands are unable to differentiate between topological classes of G4s or to selectively recognize specific G4 motifs.^3^ Therefore, achieving selectivity between different G4 topologies still remains one of the most challenging tasks in this field. Some selective compounds have been recently reported such as a coumarin–quinazolinone,^35^ a quinazoline–quinazolinone,^36^ a core-extended naphthalene diimide,^37^ squaraine dyes,^38,39^ a thiazole peptide,^31^ and anthracene-based,^40^ BODIPY-based,^41^ triarylimidazole-based,^42^ and bis(quinolinium) pyridodicarboxamide-based^43^ compounds. However, because of multiple mechanisms of action, chemical scaffolds that fall far outside the “druglike” chemical space, high molecular weights, the induction of conformational changes, and complicated multistep synthetic procedures further strategies are required to rationally design G4-interacting small optical probes as easily accessible diagnostic tools.

**Scheme 1:**
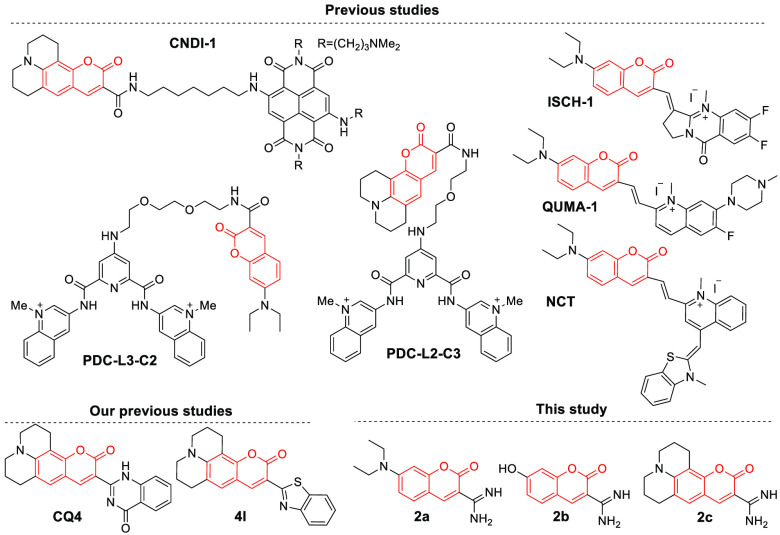
Examples of Previously Published Coumarin Derivatives Used for G4 DNA and RNA Detection *in Vitro* and in Cells.^19,35,43−47^^,^ The structures of the coumarin analogues reported by us in previous studies^35,47^ and the structures of the three derivatives used in the present study (**2a**-**c**). The coumarin scaffold is marked in red.

To detect G4s in living cells, the probe should have both high selectivity for specific G4 structures/sequences and good membrane permeability. These basic characteristics, although prerequisites, are rarely simultaneously fulfilled by the same molecular agent. Within the class of G4-probes, coumarin dyes are among the most studied compounds. In previous studies, the coumarin scaffolds were usually extensively modified through the insertion of π-extended heterocyclic aromatic motifs in order to tune both the photophysical properties and G4-binding properties to both DNA and RNA (Scheme 1).^19,35,43−47^ Even if all these molecular recognition strategies have been successfully implemented in *in vitro* models and, in some cases, in fixed and live cells, the molecular sizes of some of these newly generated probes are not ideal for membrane permeability and might therefore hinder the detection of G4 DNA in live cells.

The most-selective G4 probes show high signal in the G4-rich nucleoli,^15,19,20,35,36^ which are highly dense multifunctional domains in which ribosome biogenesis occurs with a high level of transcriptional activity involving both G4 DNA and RNA structures.^20^

Inspired by our recently published results on the use of coumarin–quinazolinone,^35^ coumarin–benzothiazole,^47^ and quinazoline–quinazolinone^15,36^ compounds, we synthesized three low-molecular-weight amidinocoumarin derivatives with druglike characteristics that differed only by the nature of their electron-donating substituents that determine their fluorescence properties (Scheme 1). By testing different G4 oligonucleotides, we found that two of these probes exhibited topology-specific G4-binding properties, but only one of them showed promising potential to detect G4 DNA in cells. This molecule displayed negligible background fluorescent signal in its unbound state. However, upon interaction with parallel G4s, a twisted intramolecular charge transfer (TICT) process opened competitive radiative relaxation pathways that led to a marked light-up fluorescence response. The resulting binding events enabled us to discriminate parallel G4s over antiparallel and non-G4 topologies through visible color changes detectable by the naked eye. The structural details of the compound’s binding interactions with parallel G4 *c-MYC* promoter structures were also assessed by 1D ^1^H NMR titration studies, showing stacking interactions to the terminal G-tetrads. Moreover, we showed that DNA synthesis in cells, studied at single-molecule level, were slowed. The slower DNA synthesis was also confirmed by a biochemical DNA polymerase stop assay that clearly showed the ability of this compound to arrest DNA synthesis prior to the G4 structure. Finally, intracellular studies indicated that this small water-soluble optical probe decrease the viability of cancer cells, and that it is capable of rapid cellular entry and nucleolar localization, thus enabling detection of G4 DNA structures in live cells.

## Results and Discussion

### Design of the Molecular Probes

All tested compounds had low molecular weights (<300 Da) and obey Lipinski’s “rule of five”, which predicts druglike properties. The compounds were synthesized in a single step (see Scheme S1 and Figures S1–S3) through Knoevenagel condensation of commercially available substituted ortho-hydroxyl benzaldehydes (**1a**-**c**) with ethyl cyanoacetate in the presence of ammonium acetate via in situ formation of 3-cyanocoumarin, which on subsequent reduction led to the formation of the desired 3-amidinocoumarin derivatives (**2a**-**c**) (Scheme 1).

### Characterization of the Compounds by Solvent-Dependent Studies

**Figure 1:**
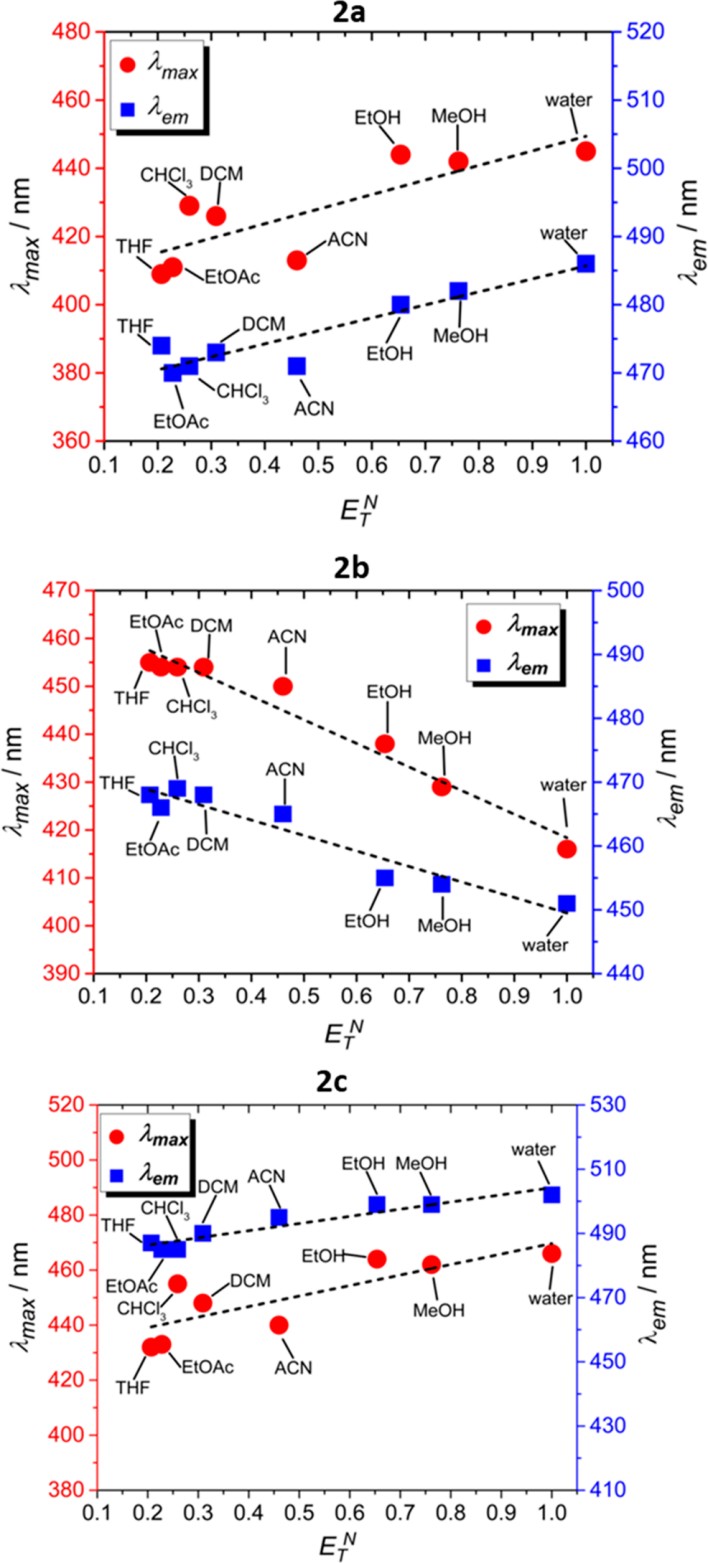
Comparison of polarity-dependent band shifts for **2a** (upper panel), **2b** (central panel), and **2c** (bottom panel) on a normalized solvent polarity scale (*E*_T_^N^).^48^

We first determined the spectroscopic properties of the coumarin derivatives **2a**–**2c** and performed solvent-dependent UV/vis absorption and emission measurements in order to determine how different solvent polarities affect the optical properties of the compounds. For these measurements, we used seven different solvents with different polarities (water, methanol (MeOH), ethanol (EtOH), acetonitrile (ACN), dichloromethane (DCM), chloroform (CHCl_3_), ethyl acetate (EtOAc), and tetrahydrofuran (THF)). By increasing the solvent polarity, the absorption maximum (λ_max_) was increased from 409 nm to 445 nm (Δλ_max_ = 36 nm) and 432 to 466 nm (*Δλ*_*max*_ = 34 nm) for **2a** and **2c**, respectively (Figures 1 and S4). Emission studies in these solvents demonstrated a shift of the *λ*_*em*_ to longer wavelengths at increasing solvent polarities. This bathochromic shift of the emission band was 474–486 nm (*Δλ*_*em*_ = 12 nm) and 487–502 nm (*Δλ*_*em*_ = 15 nm) for **2a** and **2c**, respectively (Figures 1 and S4). The plots of solvent polarity versus absorption/emission maximum indicated an overall positive solvatochromic behavior for **2a** and **2c** (Figure 1), showing that the red-shifted absorption and emission spectra of the compounds are dependent on an increased solvent polarity.

For **2b**, the solvent-dependent absorption and emission studies both indicated a negative solvatochromic effect of the compound with λ_max_ increasing from 417 nm to 455 nm (Δλ_max_ = 38 nm) and λ_em_ ranging from 451 nm to 468 nm (Δλ_em_ = 17 nm) when the solvent polarity was decreased (see Figure 1, as well as Figure S4 in the Supporting Information). These findings clearly support a greater stabilization of the first excited state of **2a** and **2c**, relative to the ground state, which is associated with the increased dipolar character of the molecules in high-polarity solvents.^48^ Conversely, the negative solvatochromism of **2b** is caused by differential stabilization of the ground and the first excited state, with the former being more energetically favorable, compared to the latter.^48^

### G4-Sensing Studies

**Figure 2:**
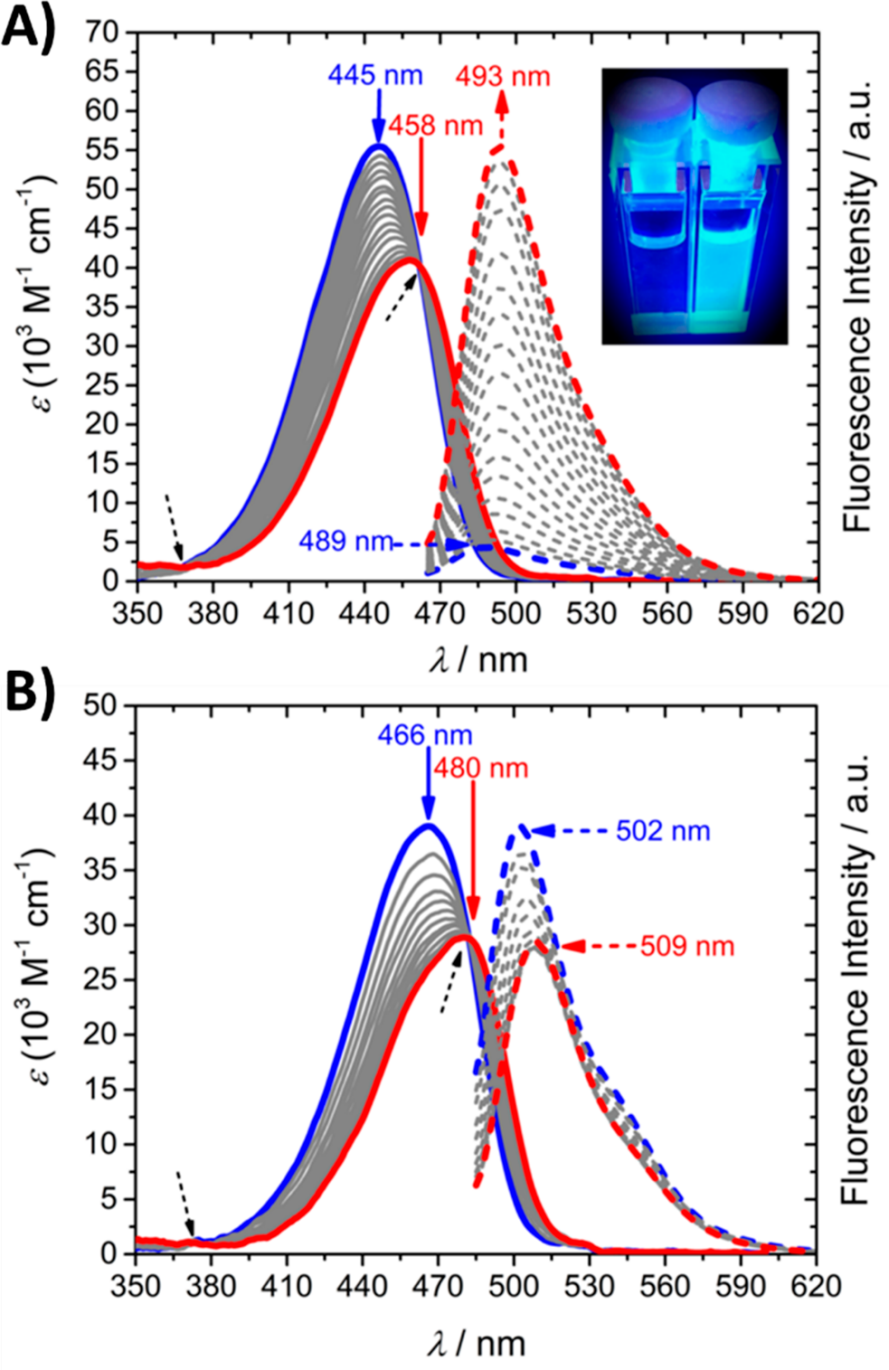
Absorption and fluorescence titrations of (A) **2a** and (B) **2c** in complex with *c*-*MYC* Pu22. The UV/vis absorption (solid lines) and fluorescence emission spectra (dashed lines) of **2a** and **2c** upon gradual addition of *c*-*MYC* Pu22 (3.0 μM or 1.5 μM **2a**/**2c**, 100 mM KCl, 50 mM Tris-buffer (pH 7.5); λ_exc__(**2a**)_ = 461 nm and λ_exc__(**2c**)_ = 482 nm). The blue and red solid lines correspond to the UV/vis spectra at (*c-MYC* Pu22/**2a**) 0.0 and 11.33 equiv and (*c-MYC* Pu22/**2c**) 0.0 and 4.66 equiv, respectively. The blue and red dashed lines correspond to the emission spectra at (*c-MYC* Pu22/**2a**) 0.0 and 23.33 equiv and (*c-MYC* Pu22/**2c**) 0.0 and 6.66 equiv, respectively. The blue and red arrows show the evolution of the binding profile at the beginning and end of the titration, respectively. The black dashed arrows show the appearance of isosbestic points. The emission intensity was normalized to the absorption maximum. The inset in panel (A) shows the color change derived from the addition of *c*-*MYC* Pu22 to **2a** as detected by the naked eye, using a 312 nm UV-lamp (3.0 μM **2a**, 34 μM *c-MYC* Pu22).

Next, we performed G4-binding studies with the different compounds to determine if they can bind and light-up upon binding to G4 DNA. In an aqueous buffer solution of high ionic strength (100 mM K^+^) without G4 DNA, the UV/vis spectra of **2a**, **2b**, and **2c** revealed well-defined intramolecular charge transfer (ICT) bands centered at 445, 416, and 466 nm, respectively (see Figures 2A and 2B, as well as Figure S5 in the Supporting Information). Upon titration of **2a** and **2c** with *c*-*MYC* Pu22, which is a well-characterized parallel G4 DNA structure (see Table S1 in the Supporting Information),^49−52^ monotonic hypochromism (*H* ≈ 26%) of the ICT band, along with a pronounced bathochromic shift of 13 and 14 nm, respectively, was observed (Figures 2A and 2B). Moreover, the appearance of well-defined isosbestic points centered at 370 and 461 nm for **2a**-*c*-*MYC* Pu22 and 373 and 482 nm for **2c**-*c*-*MYC* Pu22 indicated the formation of a structured ligand/G4 complex. These spectral modifications can be ascribed to specific short-range interactions between the hydrophobic central coumarin core that is prone to π-stacking and the in-plane G-tetrad scaffold, which decrease the π–π* energy gap and result in a red-shifted *λ*_*max*_.

Under the same experimental conditions as for **2a** and **2c**, **2b** exhibited negligible spectral changes, highlighting the inability of this compound to interact with *c*-*MYC* Pu22 (see Figure S5 in the Supporting Information). We hypothesize that the lack of G4 binding response by **2b** might arise from either the poor electron-donating character of the hydroxyl group and/or from the reduced overall net positive charge occurring at pH 7.5 (*vide infra*).

In contrast to the absorption changes, the steady-state emission spectrum of **2a**, which is almost fully quenched in its unbound state (Figure 2A, blue dashed line), showed a marked increase in the fluorescence signal along with a pronounced bathochromic effect of 4 nm upon *c*-*MYC* Pu22 titration (Figure 2A, red dashed line). This effect in the presence of *c*-*MYC* Pu22 induced a color change that could be detected by the naked eye (inset of Figure 2A).

The fluorescence intensity of compound **2c** was, in contrast to **2a**, gradually quenched (27%) and bathochromically shifted by 7 nm by the addition of *c*-*MYC* Pu22 (Figure 2B). These spectral changes for **2c** may be attributed to the increased hydrophobic interactions between the G4 nucleobases and the flat and rigid julolidine moiety, which might lead to competitive radiationless relaxation pathways. Also, the change in fluorescence in the presence of *c*-*MYC* Pu22 was not as dramatic for **2c** (∼1.5-fold) as it was for **2a** (∼14-fold) (Figure 2). Finally, similar to the UV/vis absorption data, compound **2b** did not show any changes in the emission intensity in the presence of *c*-*MYC* Pu22, thus showing no ability to detect *c*-*MYC* Pu22 (Figure S5).

**Figure 3:**
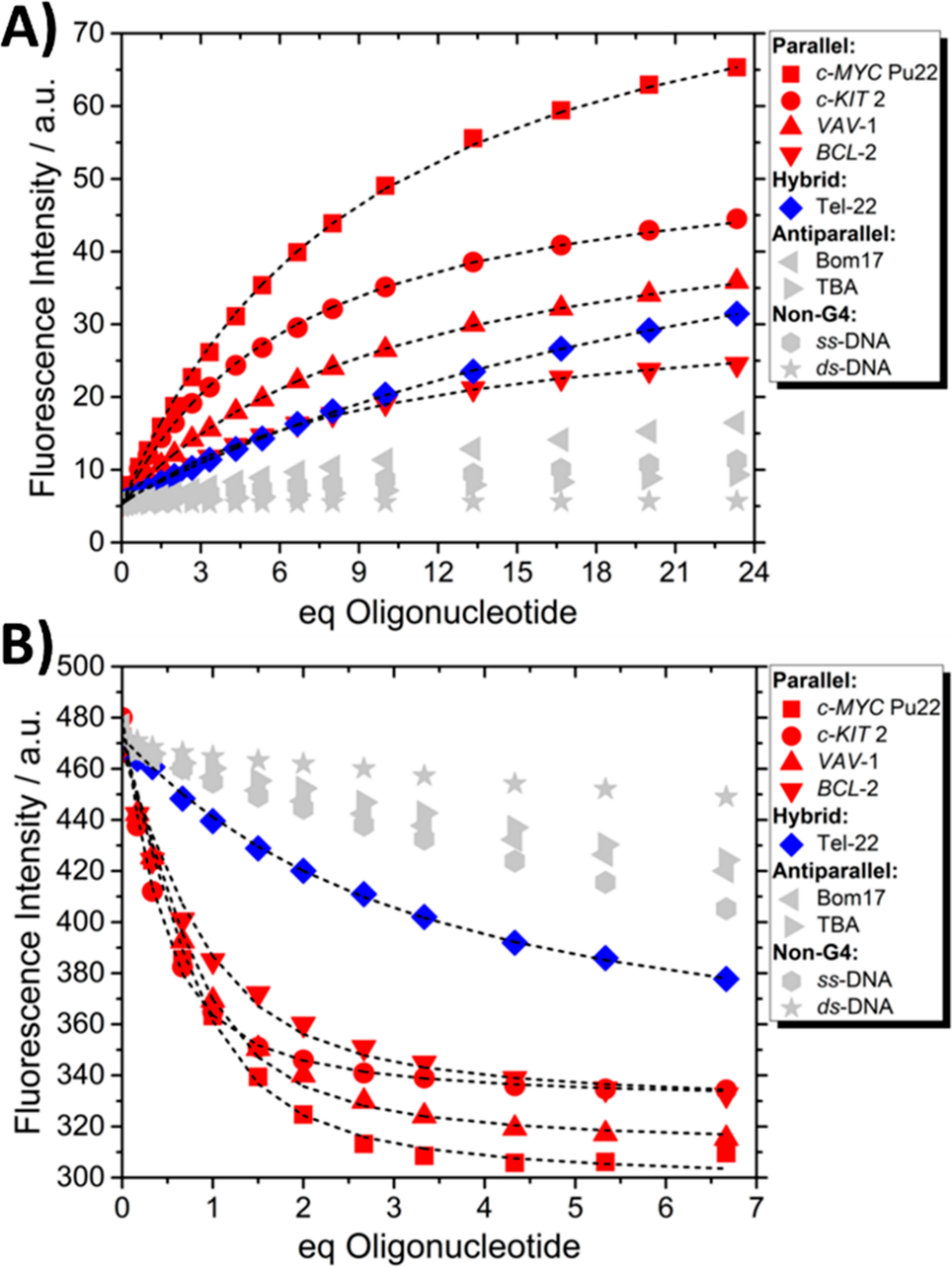
Spectrofluorimetric binding isotherms of (A) **2a** and (B) **2c** complexed with various G4 and non-G4 structures showed a clear-cut preference for parallel/hybrid G4 topologies over nonparallel and non-G4 structures. The dashed black lines correspond to a 1:1 fitting model at λ_em_ whose values are reported in Table 1 (1.5 μM **2a**/**2c**, 100 mM, KCl, 50 mM Tris-buffer (pH 7.5); λ_exc__(**2a**)_ = 461 nm, λ_exc__(**2c**)_ = 482 nm). Parallel G4 in red, hybrid G4 in blue, antiparallel G4 and non-G4 in gray.

The results above prompted us to study the recognition process of our probes toward biologically relevant natural and synthetic G4 structures forming parallel, antiparallel, and hybrid topologies (Table S1 and Figures S6–S8 in the Supporting Information), including those found in the promoter regions of the *c*-*MYC*, *c-KIT*, *BCL-2*, *VAV* genes, human telomeres (Tel-22), and TBA.^53^ As shown in Figures 3A and 3B, as well as Figures S9–S12 in the Supporting Information, both **2a** and **2c** exhibited a clear-cut binding preference for parallel G4 topologies over antiparallel and non-G4 structures. Both **2a** and **2c** showed the strongest turn-on and turn-off emission response in the presence of *c*-*MYC* Pu22, respectively. Moreover, no binding response was observed for **2b** in the presence of parallel, antiparallel, or hybrid G4 morphologies (see Figure S5). Interestingly, the selective light-up properties of **2a** toward parallel G4 structures were also detected by the naked eye (Figure S13 in the Supporting Information) or on a native nondenaturing polyacrylamide gel (Figure S14 in the Supporting Information).

The molecular recognition ability of **2a** and **2c** for parallel G4s can be attributed to their highly accessible G-quartet surfaces that provide better π-stacking platforms for the accommodation of the coumarin core. The different evolutions of fluorescence intensity observed for **2a** and **2c** in the presence of parallel G4s suggested that rigidification of **2a**’s chromophoric system upon binding the G4 leads to the increased photoluminescence quantum yield (PLQY) through a potential TICT process (Figure S15), whereas the inherent planarity of **2c** results in intense intermolecular chromophore–nucleobase interactions that are responsible for the resulting fluorescence quenching. Finally, to determine the sensitivity of **2a** for all four tested parallel G4 structures, we performed limit of detection (LOD) measurements. LOD was calculated by using the measured emission values of **2a** in complex with various concentrations of the different parallel G4 structures (see Figure S16 and Table 1). The calculated LOD values ranged from ∼128 nM to 342 nM (Table 1).

**Table 1:** Photophysical Properties of the **2a** and **2c** Compounds in the Absence (Indicated as Row **2a** and **2c**) and the Presence of G4 Structures

compound	G4 topology	λ_max_ (nm)	ε_max_ (× 10^3^ M^–1^ cm^–1^)	*H*^a^ (%)	λ_em_ (nm)	K_d_^b^ (μM)	*Φ*_*F*_^c^ (%)	LOD (nM)
**2a**	–	445	55.5	–	489	–	1.1	–
*c-MYC* Pu22	parallel	458	41.0	26.1	493	13.0	6.2	142.4
*c-KIT* 2	parallel	456	41.4	25.4	493	9.1	4.4	128.5
*VAV-*1	parallel	454	43.5	21.6	494	15.0	3.8	264.6
*BCL-*2	parallel	454	41.5	25.2	493	14.6	2.9	342.4
Tel-22	hybrid	–	–	–	494	45.2	2.9	–
**2c**	–	466	39.0	–	502	–	62.2	–
*c-MYC* Pu22	parallel	480	28.9	25.9	509	0.3	46.3	–
*c-KIT* 2	parallel	479	29.4	25.6	508	0.7	49.9	–
*VAV-*1	parallel	476	29.0	25.6	508	0.3	46.6	–
*BCL-*2	parallel	477	30.0	23	508	0.5	49.5	–
Tel-22	hybrid	472	31.4	19.5	506	3.8	53.7	–

a Percentage of hypochromic effect (*H*) on ε_max_.

b Fitting with a 1:1 binding model was obtained with Bindfit by using multiple global fitting methods (Nelder–Mead method) on the fluorimetric data.

c Coumarin 153 in ethanol (Φ_F_ = 38%) was used as the standard. PLQY are calculated at oligonucleotide/ligand ratio = 5.

Both **2a** and **2c** showed a net positive charge under our experimental conditions, as shown by structure-based calculations computed with Marvin Sketch software. The microspecies distribution of the compounds in the entire pH range is provided in Figure S17 in the Supporting Information. The number of charges at pH 7.5 was ∼0.5 for **2b** and ∼1.0 for **2a** and **2c**. However, despite the positive charge, no or very low off-target binding was detected with the negatively charged phosphate backbone of the *ss*- and *ds*-DNA. These results clearly indicate that the coordination mechanism of both coumarin derivatives toward the parallel G4 templates is not driven by nonspecific electrostatic interactions.

To further investigate the interplay between the G4 templates and fluorescence signal changes, we performed quantitative binding analysis on the fluorimetric titrations (Figures 3A and 3B and Table 1, as well as Table S2). In all cases, a global nonlinear curve fitting procedure based on a 1:1 (ligand:DNA) stoichiometry model fit well to our experimental fluorescence output data. This 1:1 stoichiometry for the **2a**:*c*-*MYC* Pu22 system was also supported by a Job’s plot where we plotted the integrated emission area as a function of the mole fractions (Figure S18).

These experiments allowed us to calculate the dissociation constant (K_d_) for **2a** and **2c** with the different DNA structures using both spectrophotometric and fluorimetric data (see Table 1, as well as Table S2). Compound **2c** complexed with parallel G4s gave the best *K*_d_ values ranging from 0.3 μM to 0.7 μM. In the presence of the hybrid telomeric G4 structure, **2c** showed a 5-to-12-fold lower binding affinity (*K*_d_ = 3.8 μM). A similar trend was observed for **2a** that exhibited *K*_d_ values ranging from 9.1 μM to 15.0 μM in the presence of the different parallel G4s. The *K*_d_ of **2a** in the presence of Tel-22 was 45.2 μM. This value was ∼5-fold higher, compared with the *K*_d_ value of the best parallel G4 sequence (*c-KIT* 2) used in this study.

These data were fully consistent with the fluorescence response, confirming the preference of the compounds for parallel G4s. The *K*_d_ values of the **2a**-*c-MYC* Pu22 and **2c**-*c-MYC*-Pu22 systems calculated using the fluorescence data were also found to be in good agreement with those calculated in our UV-vis titration experiments (see Table S2). No quantitative binding data analysis was performed for the compounds complexed with antiparallel and non-G4 structures, because the optical response of the probes, even in the presence of a large excess of biological templates, did not show a clear binding isotherm characterized by a hyperbolic saturation profile. Overall, these data outlined the ability of **2a** and **2c** to selectively target parallel G4s over antiparallel and non-G4 structures. Generally, the *K*_d_ values obtained for **2a** complexed with parallel G4 structures are higher, compared to those reported for other selective parallel G4 ligands (*K*_d_ values between 10 and 0.1 μM).^31,35,37,40−42^ On the other hand, **2c** showed excellent parallel G4-interactive binding properties that match some of the best topology selective G4-binders reported so far.^31,35,37,40−42^

Because both **2a** and **2c** featured the same chromophoric system and number of positive charges, we speculate that their different binding strengths are due to the nature of the electron-donating substituents. The higher binding affinities demonstrated for the **2c**-G4 DNA complexes compared to the **2a**-G4 DNA complexes are likely linked to additional hydrophobic interactions in combination with a reduced entropic loss upon binding of **2c**, because the julolidine moiety already is locked in a flat conformation.

Unfortunately, the fluorescence quenching of **2c** bound to parallel G4s may prevent its application as a cellular fluorescent reporter. Therefore, we focused our attention on compound **2a** that showed optical properties suited for *in-cellulo* studies.

### **2a** Stabilizes G4 Structures

**Figure 4:**
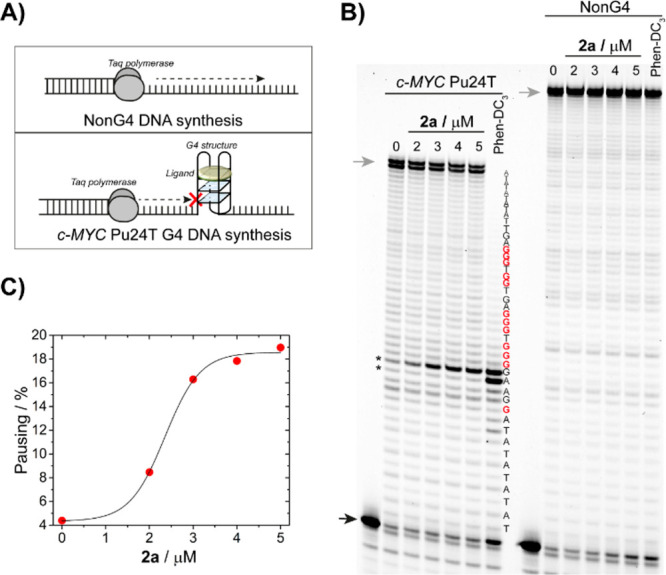
Compound **2a** stabilizes the *c-MYC* Pu24T structure and selectively inhibits DNA synthesis by Taq-DNA polymerase during DNA replication, while DNA replication on the non-G4 DNA template is not affected. (A) Schematic representation of the Taq polymerase stop assay with either a non-G4 template or the *c-MYC* Pu24T G4 DNA template. (B) Taq-DNA polymerase stop assay with **2a** using *c-MYC* Pu24T and non-G4 DNA templates. The concentration of **2a** is indicated above each lane. Phen-DC_3_ (0.32 μM) was used as the G4-stabilizing reference compound. Black arrows indicate nonextended primer, gray arrows indicate full-length products, and asterisks indicate pausing sites. (C) Quantification of pausing effects of **2a** on the *c-MYC* Pu24T G4 template.

To determine if **2a** not only selectively binds and lights up G4s, but also stabilizes these structures, we performed a Taq-DNA polymerase stop assay (Figure 4A). In this assay, we determined the stabilization and selectivity by comparing the effect of the compound on DNA synthesis of a non-G4 or *c-MYC* G4 template. The reaction products were loaded onto a denaturing polyacrylamide gel, and progression and pausing of the DNA polymerase was determined at single nucleotide resolution.^15,36^ By increasing the **2a** concentration, we found increased amounts of replication pausing one nucleotide before the first G-tract on the G4 template as well as decreased amounts of full-length products (Figures 4B and 4C). This showed that **2a** inhibits DNA synthesis of Taq-DNA polymerase by selectively stabilizing the *c-MYC* G4 template in a concentration-dependent manner. The non-G4 template was not affected by **2a**, thus supporting the selectivity of our probe for *c-MYC* G4. The selective stabilization of the G4 structure and pausing before the first G-tract by **2a** was confirmed by using the well-known G4-stabilizer Phen-DC_3_ as a control.

### **2a** Binds by Stacking on the Terminal G-Tetrad

Next, the effect of **2a** on the conformation of parallel G4s was investigated by electronic circular dichroism (ECD) measurements. The ECD spectrum of parallel G4s is characterized by typical positive and negative dichroic signals at 265 and 240 nm, respectively (see Figure S19 in the Supporting Information).^53^ The gradual addition of **2a** to the *c-MYC* Pu22 or *c-KIT* 2 solution did not induce changes in either the magnitude or the shape of the bands. These data suggest that **2a** can exert its topology-specific sensing/stabilization function without inducing topological transitions on the G4 template.

**Figure 5:**
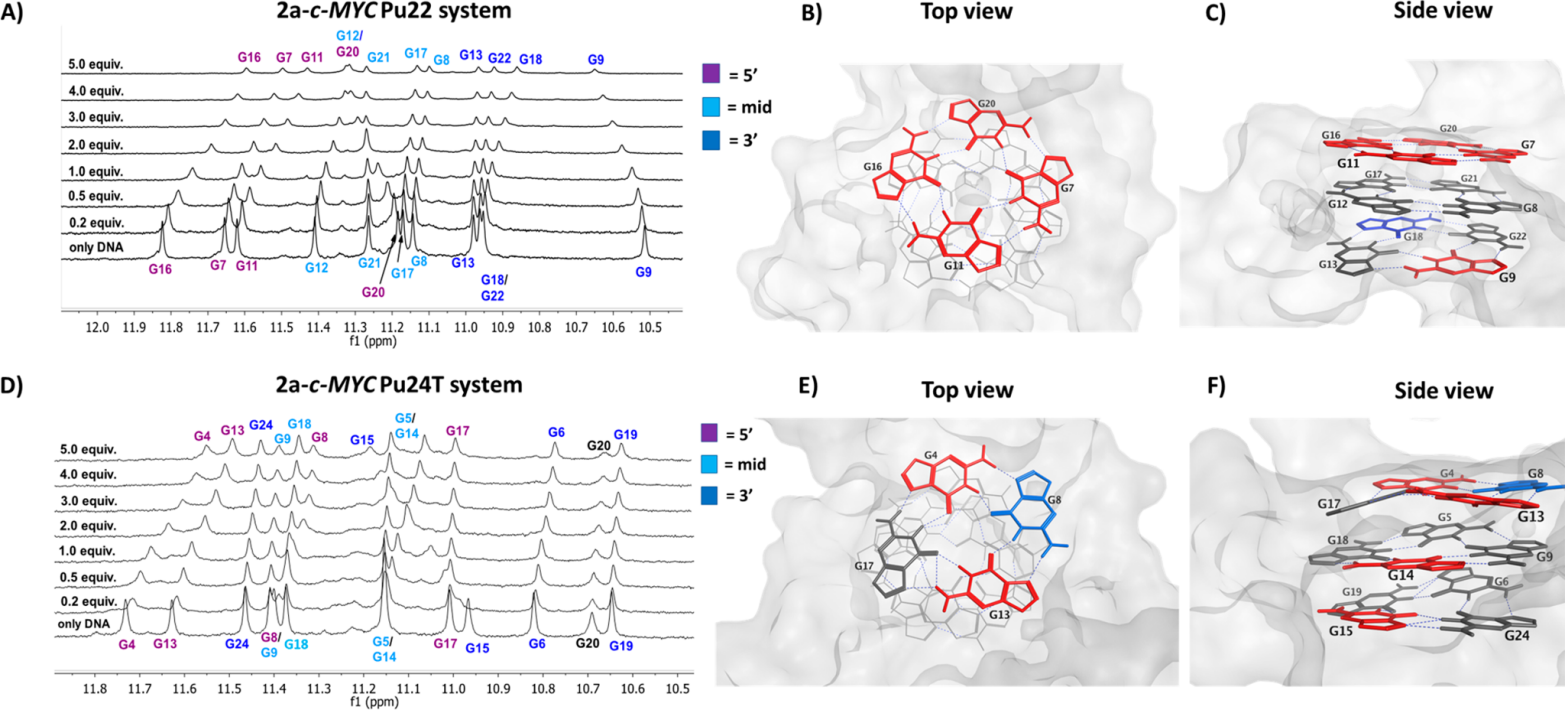
^1^H NMR (850 MHz) titrations for (A) *c-MYC* Pu22 and (D) *c-MYC* Pu24T complexed with **2a**. The initial G4 concentration was 90 μM, and **2a** was then added so that the last addition corresponded to a total molar ratio of G4 DNA:**2a** of 1:5. The guanines involved in the formation of the G4 structure are color coded. G20 is not involved in the G4 structure.^54^ Graphical representation of (B, C) *c-MYC* Pu22 (PDB: 5W77) and (E, F)*c-MYC* Pu24T (PDB: 2MGN) generated in MOE. The top images are viewed from the 5′-side of the G4-structures. The guanines in the quadruplex structures are color-coded, based on how affected their imino protons were throughout the titration experiments. Red: strong changes, blue: moderate changes, and gray: no or very weak changes.

In order to determine the site localization of our probe on the *c-MYC* G4 templates (*c-MYC* Pu22 and *c-MYC* Pu24T), we performed 1D ^1^H NMR titration studies (see Figures 5A and 5D). In these experiments, the G4 DNA was titrated with increasing concentrations of **2a** and the changes occurring in the imino protons of the G4 templates were analyzed.^32,49,54^ In the *c-MYC* Pu22-**2a** system, a clear shift for all the imino protons belonging to the 5′-quartet was observed, indicating specific interactions between **2a** and the 5′-end of the G4 (Figure 5A). No or very weak changes of the imino protons belonging to the central quartet were observed throughout the titration, thus ruling out their involvement in the complexation mechanism. Both the G9 and G18 residues located at the 3′-end displayed evident changes, whereas G13 and G22 remained almost unaffected. An explanation for this observation could be that **2a** can only partially access the 3′-end, thus giving rise to specific interactions only with the G9 and G18 sites (Figure 5A).

An analogous experiment performed with *c-MYC* Pu24T, a sequence that features the same 5′-G-tetrad/flanking residue of *c-MYC* Pu22 but a considerably different 3′-terminal G-tetrad end and flanking sequence,^54^ showed pronounced structural changes at the 5′-quartet of the G4 template, thus supporting the ability of **2a** to coordinate the 5′-end of the *c-MYC* G4s (Figure 5D). Interestingly, G15 in the 3′ quartet alone was affected as well as G5 or G14 in the central quartet. To better visualize which of the guanines had the major contribution in the complexation event with **2a**, a schematic illustration of *c-MYC* Pu22 (see Figures 5B and 5C) and *c-MYC* Pu24T (Figures 5E and 5F) was generated using the Molecular Operating Environment (MOE) software. In these illustrations, the guanines in the G4 scaffolds were color-coded, depending on how affected they were throughout the titration experiments.

To conclude, the chemical shifts changes of the imino protons upon **2a** binding suggest that the coumarin derivative coordinates the *c-MYC* G4 templates through an end-stacking mode that mainly involve both the 5′- and 3′-ends.

In order to further examine the terminal binding of **2a** with the *c-MYC* G4 template, we performed a fluorescence displacement competition assay in the presence of Phen-DC_3_. The resolved solution structure of Phen-DC_3_ bound to the G4 parallel sequence derived from the *c-MYC* promoter demonstrates the highest-affinity binding site at the 5′-end and a second binding event at the terminal 3′-end, thus showing that Phen-DC_3_ is a G4 end-stacking molecule.^54^ Because **2a** binds with specificity to parallel G4s, exhibiting association-induced emission, we hypothesized that if competition for the same binding sites occurs between **2a** and Phen-DC_3_, a decrease in the fluorescence intensity should be observed (Figure S20A in the Supporting Information). Indeed, we found that the fluorescence signal of the **2a**-*c-MYC* Pu22 system was efficiently quenched and blue-shifted by the addition of increasing concentrations of Phen-DC_3_ (Figure S20B in the Supporting Information). These results highlight the strong competitive behavior of the two molecules for the same binding sites and suggest that **2a** binds to *c-MYC* G4 by stacking on the terminal G-tetrad.

To further examine the terminal stacking binding mode of **2a** to parallel G4 structures, we used another G4 structure, *c-MYC* sG4, which has an identical internal sequence as *c-MYC* Pu22 except for the 5′ and 3′ terminal flanking regions, but still forms a parallel G4 structure (*c-MYC* Pu22 = 5′-TGA**GGGTGGGTAGGGTGGG**TAA-3′; *c-MYC* sG4 = 5′-**GGGTGGGTAGGGTGGG**-3′).^35,38^ By lacking these flanking regions, we hypothesized that *c-MYC* sG4 would provide better π-stacking possibilities for the planar conformation of the coumarin core (Table S1). Indeed, titration of **2a** with *c-MYC* sG4 induced an ∼20-fold fluorescence enhancement, which was 6-fold higher, compared to that of **2a** complexed with *c-MYC* Pu22, and the *K*_d_ value was 12.6 μM (see Figures S21A and S21Bin the Supporting Information). Furthermore, the light-up ability of **2a** titrated in the presence of the competitive complementary *c-MYC* sG4 C-rich sequence (sC4 = 5′-CCCACCCTACCCACCC-3′) and duplex DNA was nearly unchanged (∼15-fold emission enhancement) (Figure S21C). These results highlight the high selectivity of **2a** for parallel G4 structures in a complex and highly competitive environment.

Finally, we also verified the selectivity of **2a** for parallel G4s by performing competitive PAGE studies (Figure S21D in the Supporting Information). In this assay, we used *c-MYC* Pu22 as the target template (20 μM) in the absence or presence of 100 μM of the competitive antiparallel G4 TBA, GC-rich *ds*-DNA (formed by annealing *c-MYC* Pu22 with its complementary strand), or self-complementary *ds*-DNA. **2a** was able to impart parallel G4 specificity even in the presence of a 5-fold excess of these nonparallel G4 structures (Figure S21D). Together our *in vitro* data show that **2a** is a selective light-up parallel G4 binder.

### Cellular Imaging Reveals **2a** Uptake in Live Cells

**Figure 6:**
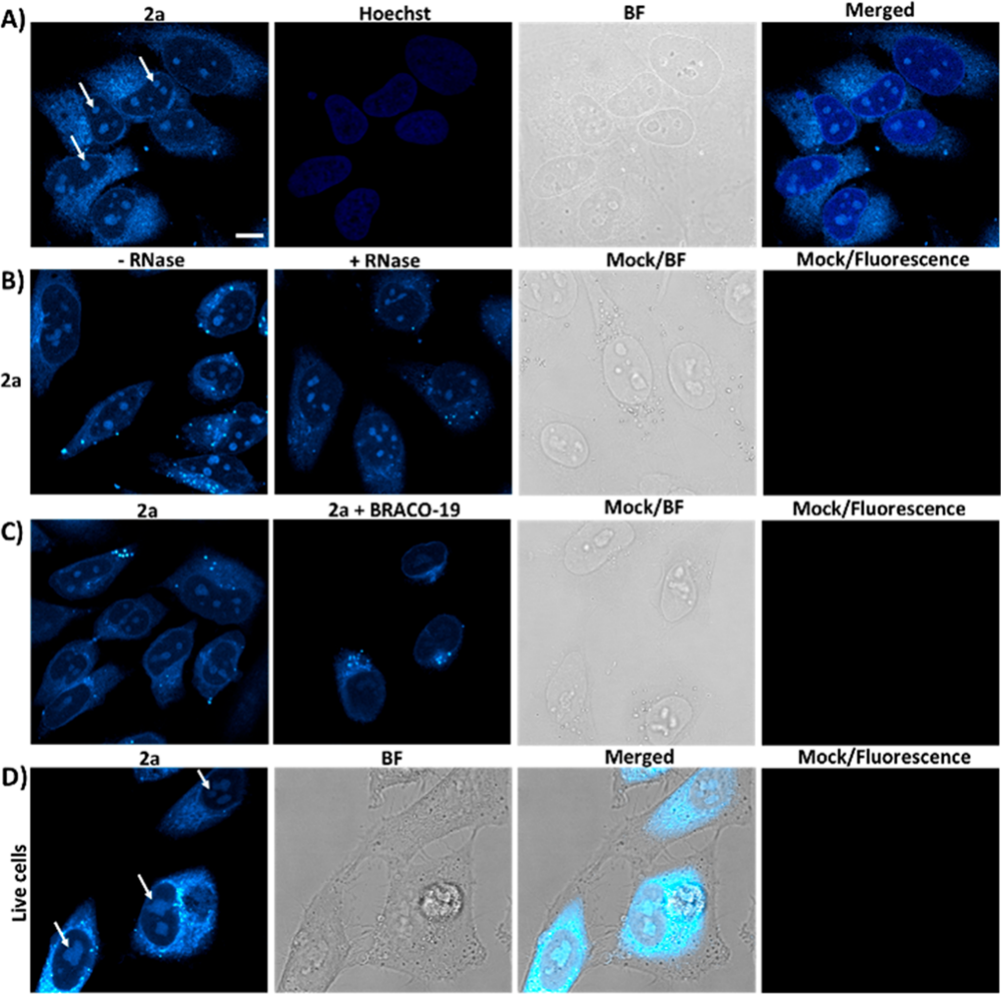
Confocal fluorescence images of HeLa cells. (A) Fixed HeLa cells stained with **2a** (20 μM), Hoechst 33342 (1 μM), and corresponding bright-field (BF) and merged images. The white arrows indicate the **2a**-mediated nucleoli staining. (B) Fluorescence images of fixed HeLa cells stained with **2a** (20 μM) without and with RNase treatment. The BF and fluorescence images for untreated cells show the absence of any autofluorescence signal. (C) Fluorescence displacement assay with **2a** (20 μM) in the absence or presence of BRACO-19 (20 μM). The BF and fluorescence images for cells treated only with BRACO-19 (20 μM) show the absence of any BRACO-19-associated fluorescence signal. (D) Fluorescence, BF, and overlay images of live HeLa cells stained with **2a** (10 μM) for 10 min. The white arrows indicate the **2a**-mediated nucleoli staining in living cells. The fluorescence images for untreated cells show the absence of any autofluorescence signal. Scale bar = 10 μm. Experimental settings (A–D): A 405 nm diode laser was used for Hoechst 33342 (λ_exc_ = 405 nm, λ_em_ = 410–445 nm), and an argon laser (λ_exc_ = 458 nm, λ_em_ = 470–700 nm) was used for **2a** excitation.

To determine if **2a** can be used to visualize G4 structures in cells, we used confocal laser scanning microscopy. Fixed HeLa cells treated with 20 μM **2a** revealed intense fluorescence signals both in the extranuclear cellular regions and in the subnuclear G4-rich compartments whose appearance is compatible with that of nucleoli (Figure 6A).^15,35,36,55−58^ The extranuclear signal suggests lysosomal accumulation of **2a**, unfortunately a common feature for many G4 probes, even those that operate at single-molecule level.^15,23,35,36,57,58^

Following the *in vitro* evidence for **2a**’s high selectivity for parallel DNA G4 structures, we pretreated the cells with RNase to confirm the nature of the main binding target of the compound (see Figure 6B, as well as Figure S22 in the Supporting Information). RNase treatment did not modify **2a** nucleolar staining, thus indicating the ability of **2a** to preferentially target DNA G4 structures.^35,36^ Because DNase treatment does not affect the nucleolar compartments,^22,55^ we validated the G4-binding ability of **2a** in the nucleolar sites through a competitive binding assay using the well-known G4 binder BRACO-19^36,59^ (Figure 6C). In the presence of BRACO-19, the **2a**-associated staining was strongly reduced (Figure S23 in the Supporting Information). These results suggest that BRACO-19 can compete for **2a**’s binding sites.

Finally, we asked if **2a** can reach the subnuclear G4 compartments of living cells. As shown in Figure 6D, **2a** clearly stained the nucleoli after a 10 min incubation, thus confirming the results obtained in fixed cells. The observed signal was specific to **2a**, because, under the same experimental conditions used to image **2a**, no autofluorescence signal from endogenous cellular chromophores/components was detected.

### **2a** Impaired DNA Replication and Reduced Cell Viability in HeLa Cells

**Figure 7:**
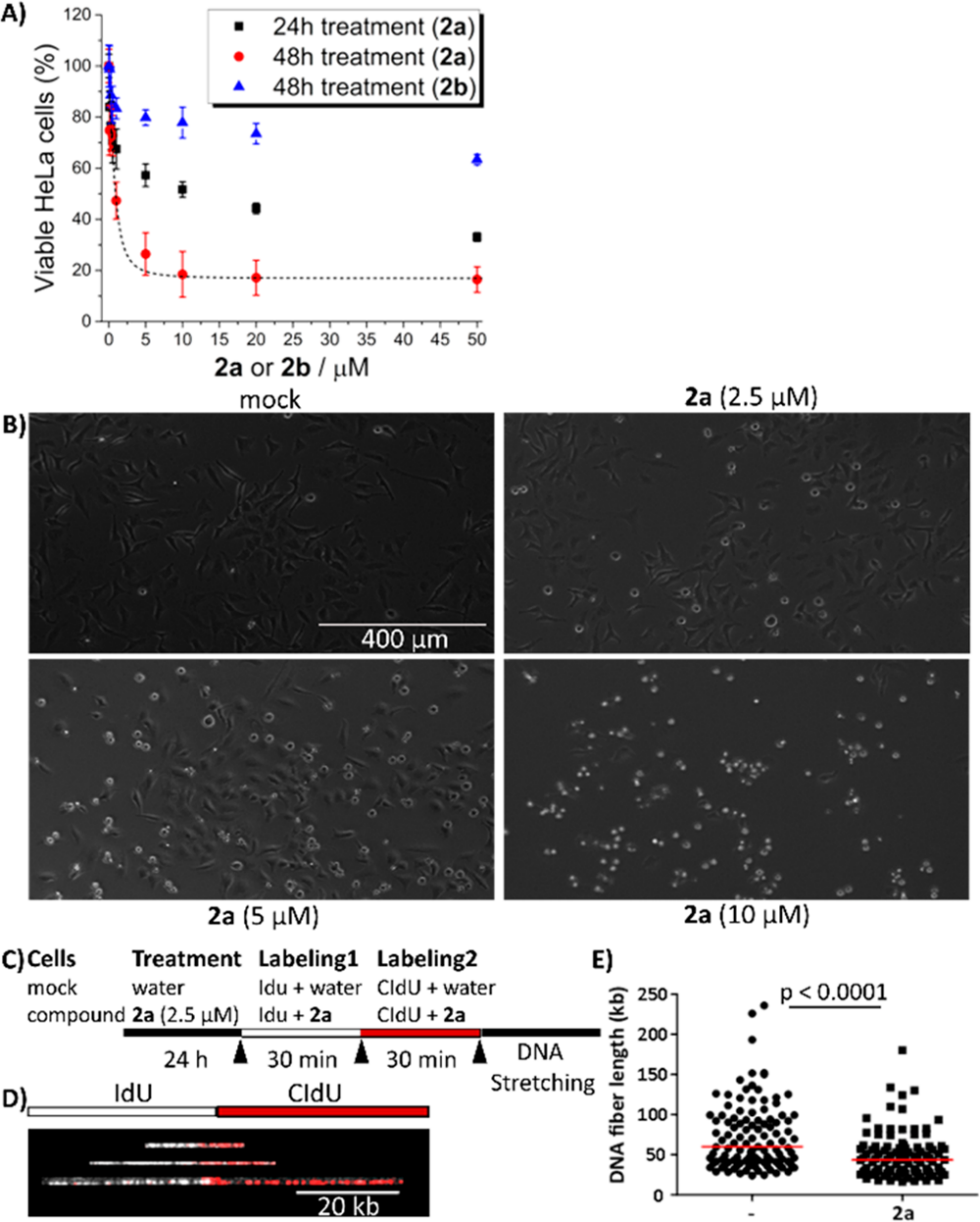
HeLa cells are sensitive to **2a** resulting in impaired DNA replication. (A) Cell viability assay of HeLa cells treated with **2a** (24 and 48 h) or **2b** (48 h). Data are shown as the mean ± SD, *n* = 3. (B) Bright-field images of untreated or treated HeLa cells with 0 (mock), 2.5, 5.0, or 10.0 μM **2a** for 24 h. (C) Schematic of the DNA fiber analysis. (D) Representative images of replication tracks of different lengths showing IdU labels (white) flanked by CIdU labels (red). (E) Quantification of the DNA fiber length (kb) in mock cells (−) versus treated (**2a**). Data represent the populations of individual DNA fibers for each condition (109 for control and 100 for treatment). Statistical analysis was performed using the nonparametric Mann–Whitney U test, and medians and *p*-values are indicated.

HeLa cells have increased amounts of G4 DNA structures compared to noncancerous cells.^15,16^ Therefore, we investigated whether G4 stabilization by **2a** affects HeLa cell viability. For these experiments, we treated the cells with either **2a** or **2b**, which served as a non-G4 control compound, and assessed the metabolic activity of viable cells with the MTT cell viability assay. After treating HeLa cells with **2a** for 48 h, there was a concentration-dependent reduction in cell viability with an IC_50_ ≈ 1.0 μM (Figure 7A). In contrast, treatment of HeLa cells with **2b** had little effect on cell viability with an estimated IC_50_ > 50 μM. These data suggest that the higher toxicity exerted by **2a**, compared with that exerted by **2b**, might be due to the ability of **2a** to bind and stabilize DNA G4s. The effect of **2a** on cell viability was dependent on the period of exposure of the cells to the compound (Figure 7A), and the 24 h treatment with **2a** showed a reduced cytotoxic effect compared to 48 h. We also monitored the cellular morphology of HeLa cells after 24 h treatment with various concentrations of **2a** (Figure 7B). Similar to the results of the MTT viability assay, we observed increased rounding morphology of HeLa cell, which is an indication of cytotoxicity,^60^ as the concentration of **2a** increased.

One explanation for the impaired cell viability might be altered DNA replication.^61^ We tested this hypothesis by visualizing and measuring newly replicated DNA molecules by performing DNA fiber analysis in HeLa cells (see Figures 7C–E, as well as Figure S24 in the Supporting Information).^15^ The mean length of the newly replicated DNA molecules in **2a**-treated cells was significantly shorter than that of the untreated cells (*p* < 0.0001), indicating that **2a** affects the rate of DNA synthesis (Figure 7E).^15^ The decreased replication rate might result from slower replication fork progression, due to **2a**’s ability to stabilize G4s.

## Conclusions

With the goal of developing more accurate and efficient G4-ligands, we investigated the G4-binding ability of three coumarin derivatives having different electron-donating characteristics. By using various biophysical and biochemical methods, we have generated structure–activity relationships that provide valuable information for the design of optical sensors that use distinct fluorescence mechanisms (e.g., TICT) to signal the presence of parallel G4 topologies. In particular, we focused our attention on a small water-soluble fluorescent light-up probe capable of specifically targeting parallel G4 structures over antiparallel and non-G4 structures. This sensor selectively signaled the presence of parallel G4 morphologies via a TICT mechanism. Its striking optical changes enabled naked-eye discrimination between different G4 topologies and non-G4 structures. Furthermore, its recognition ability was very selective for parallel G4 structures even in the presence of highly competitive *ds*-DNA or complementary C-rich DNA. The structural origin of the compound’s binding interactions with parallel G4 *c-MYC* promoter structures was assessed by 1D ^1^H NMR titration studies and showed the ability of this ligand to coordinate the G4 structures via an end-stacking binding mode. Besides its enticing quadruplex interacting and optical properties, this fluorescent sensor was also able to selectively stabilize the G4 template and inhibit DNA synthesis *in vitro*. Confocal fluorescence images of this probe in both fixed and live HeLa cells showed efficient cell permeability and nucleolar DNA G4 binding. Intracellular studies indicated that this compound decreased the viability of cancer cells and reduced DNA replication speed through a possible G4-dependent mechanism. We believe that the low molecular weight and straightforward synthesis of the evaluated compounds combined with the presented findings will be useful for the design of specific bioprobes with optimized optical performances and G4 binding parameters to be used in *in vivo* models.

## Experimental Section

### Materials

All reagents, solvents, chemicals, and biological templates were purchased from Sigma–Aldrich or Eurofins Genomics and used without further modifications unless otherwise stated. The stock solutions of all synthesized G4-binding compounds were prepared in DMSO at a concentration of 0.5 mM unless otherwise stated. Compound **2a** was also prepared in Milli-Q water at a concentration of 0.25 mM for cellular studies. The final concentration of DMSO in all the DNA-based assays was kept below 2.0% (v/v).

### G4 Folding

The oligonucleotides were diluted with ultrapure water to a concentration of 1 mM and stored at 5 °C. The exact oligonucleotide concentration was determined by UV-vis spectroscopy using the molar extinction coefficients (ε_260_) provided in Table S1 and calculated using the oligo analyzer tool on the IDT Web site. The oligonucleotides were heated at 95 °C for 5 min in the presence of 100 mM KCl and then slowly allowed to reach RT overnight.

### UV-vis Absorption and Steady-State Emission Measurements

UV/vis absorption spectra were recorded on a T90+ UV/vis spectrometer (PG Instruments, Ltd.) with a spectral bandwidth of 1 nm. Steady-state fluorescence spectra were recorded on a Jasco FP-6500 spectrofluorometer equipped with the Jasco Peltier-type temperature controller (Model ETC2736). The slit width of both monochromators was 3 nm. Relative fluorescence quantum yields (Φ_F_) were determined using Coumarin 153 as the reference (Φ_F_ = 0.38 in EtOH) keeping the optical density (OD) < 0.1. Φ_F_ was calculated by using the excitation wavelengths at 420 or 440 nm according to the following equation (eq 1):
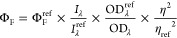
1where *I* is the integrated emission area, OD the optical density at the excitation wavelength, and η the refractive index of the solutions.

### Electronic Circular Dichroism (ECD) Measurements

ECD spectra were measured with a Jasco Model J-720 spectropolarimeter that was equipped with the Jasco Peltier-type temperature controller (Model PTC-423L) and are presented as the sums of three accumulations. ECD spectra were obtained in the range of 220–490 nm with 2 μM G4 DNA. Appropriate references were subtracted from the obtained ECD spectra. All optical measurements were performed in quartz dual path length cuvettes.

### Spectrophotometric and Fluorimetric Titrations

A 3.0 μM and 1.5 μM solution of **2a**–**2c** for UV-vis and fluorescence studies, respectively, was prepared by diluting the stock solution in a suitable amount of buffer (1.2% or 0.6% DMSO, 100 mM KCl, and 50.0 mM Tris-buffer (pH 7.5)). The freshly prepared **2a**–**2c** solutions were titrated with the folded oligonucleotide solution and allowed to equilibrate for several minutes before recording the UV/vis or emission spectra. The concentration in each experiment was optimized to have OD < 0.15 in order to avoid reabsorption of the fluorescence emission. The excitation wavelength was set at the isosbestic point (i.e., λ_exc_**2a** = 461 nm, λ_exc_**2c** = 482 nm) to avoid changes in the OD. Compound **2b** was excited at λ_exc_**2b** = 417 nm. All of the emission spectra were baseline-corrected.

### Nonlinear Global Fitting of the Binding Isotherms

All of the data were corrected for the dilution upon titration. Binding constants were obtained with Bindfit using multiple global fitting methods (Nelder–Mead method) with the fluorescence data in the range of 475–555 nm and 490–545 nm for **2a** and **2c**, respectively.^62,63^ Dilution corrections were included in the fitting option. In order to ensure that we found the minima in the fitting analyses, all of the fittings were confirmed with three different start values.

### Limit of Detection

LOD experiments were performed by plotting the changes in the emission maximum (λ_em_) as a function of parallel G4 concentration. LOD was calculated according to the following equation:
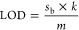
2where *s*_b_ is the standard deviation calculated out of 10 independent measurements of a blank solution, *k* is 3 according to IUPAC recommendations, and *m* is the slope obtained from the linear fitting (*I*_(λ_em_)_ vs [parallel G4]).

### Job’s Plot

The total concentration of **2a** and *c-MYC* Pu22 was held constant (5 μM) while varying the relative proportions of **2a** and *c-MYC* Pu22. The units on the *x* axis morph from concentration to mole fraction of **2a** and *c-MYC* Pu22 (χ_**2a**_ = [**2a**]/[**2a**] + [*c-MYC* Pu22]).

### Nuclear Magnetic Resonance (NMR) Titrations

The G4 DNA stock solutions (180 μL) were prepared by folding 100 μM *c-MYC* Pu24T or *c-MYC* Pu22 in 10 mM potassium phosphate buffer (pH 7.4) and 35 mM KCl by heating to 95 °C and cooling to ambient temperature on ice. Then, D_2_O (20 μL) was added to the DNA stock solutions, yielding a final DNA concentration of 90 μM. NMR samples were prepared by sequential addition of **2a** from 5 mM DMSO-*d*_6_ stock solutions to 200 μL of the DNA solution, which was then transferred to 3 mm NMR tubes. Control samples with *c-MYC* Pu24T and *c-MYC* Pu22 with and without 10% DMSO-*d*_6_ were also recorded to verify that DMSO did not have a significant effect on the G4 structures. All spectra were recorded at 298 K on a Bruker 850 MHz Avance III HD spectrometer equipped with a 5 mm TCI cryoprobe. Excitation sculpting was used in the 1D ^1^H NMR experiments, and 256 scans were recorded. Processing of the spectra was performed in MestreNova 10.0.2. available at http://www.chemcomp.com

### Polyacrylamide Gel Electrophoresis (PAGE)

PAGE was conducted on 20% native gels in TBE buffer supplemented with 100 mM KCl. Oligonucleotides were heated at 95 °C for 5 min in the presence of 100 mM KCl and then slowly allowed to reach RT overnight. The oligonucleotides were then loaded on the gel and electrophoresis was run at 80 V for 120 min at RT. After electrophoresis, the gel was incubated with **2a** (5 μM) and, where indicated, also with Thiazole Orange (TO, 5 μM) for 30 min and rinsed with TBE buffer. Visualization was performed on a Typhoon Scanner 9200 (GE Healthcare), using an excitation wavelength of 457 nm.

### DNA Polymerase Stop Assay

The DNA polymerase stop assay was performed as described previously.^64^ Briefly, reaction mixtures containing 40 nM template DNA (G4 or non-G4) annealed to a TET-labeled primer were incubated with 0, 2, 3, 4, or 5 μM **2a** in the presence of 50 mM KCl. Phen-DC_3_ (0.32 μM) was used as the reference G4 compound. Control reactions contained 2% DMSO instead of compound. Reactions were incubated with 0.625 U/μL Taq-DNA polymerase and incubated for 30 min at 50 °C. UV-vis spectroscopy was used to monitor the intrinsic thermal stability of **2a** at 95 °C (Figure S25 in the Supporting Information). The oligonucleotide sequences used are listed below:

#### Primer 5′-3′

TET-TGAAAACATTATTAATGGCGTCGAGCGTCCG.

#### *c-MYC* Pu24T 5′-3′

ATATATATATTGAGGGTGGTGAGGGTGGGGAAGGATATATATATCGGACGCTCGACGCCATTAATAATGTTTTCA.

#### NonG4 5′-3′

GAGACCATTCAAAAGGATAATGTTTGTCATTTAGTATATGCCCCTGCTCGTCTTCCCTTCTCCGGACGCTCGACGCCATTAATAATGTTTTCA.

### Cell Viability

HeLa cells (4 × 10^3^ cells/well) were seeded in DMEM high glucose media (Gibco) supplemented with 10% fetal bovine serum and penicillin-streptomycin on 96-well plates the day before the treatment. Compounds (**2a** or **2b**) were dissolved in media at the indicated concentrations and added to the cells. At 24 or 48 h after treatment, MTT (5 mg mL^–1^ stock) was added to each well and the cells were incubated at 37 °C for three additional hours. DMSO was then added to each well and incubated on a shaker for 15 min. Absorbance at 590 nm was recorded using a Synergy 200 microplate reader. The data were normalized to the control and plotted with means and standard errors. Images of cells after treating with **2a** for 24 h were acquired using the EVOS FL Cell Imaging System (Life Technologies).

### DNA Fiber Analysis for HeLa Cells

Asynchronous HeLa cells at 70% confluence were seeded at 1 × 10^5^ cells for 24 h prior to the 24 h treatment with **2a** (2.5 μM) or an equivalent volume of water (control cells). Pulse-labeling of cells with IdU and CIdU and subsequent immunostaining of DNA fibers were performed as previously described.^15^ DNA fibers were visualized using a Leica Thunder Widefield microscope, and images were captured randomly from different fields that contained untangled fibers. Only fibers containing IdU labels flanked by CIdU labels with intact *ss*-DNA ends were selected for analysis using the LASX (Leica) and ImageJ software packages. A minimum of 100 individual DNA fibers were measured for each experimental condition in two independent experiments. Measurements were made in micrometers and converted to kilobases using a conversion factor for the length of a labeled track of 1 μm corresponding to roughly 2 kb.

### Fluorescence Microscopy

HeLa cells (a cervical cancer cell line) were cultured at 37 °C in 5% CO_2_ in DMEM high glucose media (Gibco) supplemented with 10% fetal bovine serum and penicillin-streptomycin. For live-cell imaging, cells were treated with **2a** (10 μM) for 10 min before performing microscopy. For fixed cell imaging, cells were fixed in 2% paraformaldehyde for 10 min and permeabilized with PBST (phosphate-buffered saline supplemented with 0.1% Triton X-100). Fixed cells were treated with **2a** (20 μM) for 30 min at RT. The HeLa cell nuclei were visualized with Hoechst 33342 (1 μM). For the fluorescence competition assay, **2a** (20 μM) was incubated with BRACO-19 (20 μM) for 30 min at RT. For the RNA degradation assay, 1 mg mL^–1^ RNase A (Thermo Fisher) was used and samples were preincubated with RNase A for 2 h at 37 °C prior to **2a** treatment. Images were acquired on a confocal microscope Leica SP8 FALCON (Fast Life Time Contrast) using an HC PL APO 63*x*/1.20 Water motCORR CS2 objective. Intensity projections of Z-stack images were used for data presentation. Quantitative data analysis was performed by selecting the regions of interest and measuring the average fluorescence signal from the selected areas. All data were processed with the ImageJ software available at https://imagej.nih.gov/ij/.

## Supporting Information Available

Optical studies, G4 characterization, G4-interactive binding studies, limit of detection, structure-based calculations, Job’s plot, ECD spectra, displacement assays, G-tetrad selectivity, cellular quantification and synthesis of the compounds (PDF)
