# Supplementary material for: A Minimalistic Coumarin Turn-On Probe for Selective Recognition of Parallel G-Quadruplex DNA Structures
Source: ACS Chem Biol. 2021 Jul 30;16(8):1365–76. doi: 10.1021/acschembio.1c00134 (PMC8397291; doi:10.1021/acschembio.1c00134)
Supplement: Supplementary file 1 — cb1c00134_si_001.pdf [file cb1c00134_si_001.pdf]

Supporting Information

A minimalistic coumarin turn-on probe for selective recognition of parallel G-quadruplex DNA structures

Marco Deiana,[1] Ikenna Obi,[1] Måns Andreasson,[2] Shanmugam Tamilselvi,[1] Karam Chand,[2] Erik Chorell[*2] and Nasim Sabouri[*1]

[1] Department of Medical Biochemistry and Biophysics, Umeå University, 90187 Umeå, Sweden

[2] Department of Chemistry, Umeå University, 90187 Umeå, Sweden

* corresponding authors

Email: erik.chorell@umu.se

Email: nasim.sabouri@umu.se

Table of contents:

Synthesis……………………………………………………………………………………...pS3

Solvent-dependent optical studies……………………………………………………………pS8

Spectrophotometric and fluorimetric titration of 2b in the presence of G4 structures……………...pS9

G4 characterization…………………………………………………………………………pS10

Binding of 2a to various G4 and non-G4 structures………………………………………pS12

Binding of 2c to various G4 and non-G4 structures………………………………………pS14

Parallel G4 recognition mediated by 2a……………………………………………………..pS16

Enhancement of the fluorescence intensity of 2a in viscous buffer supports the TICT process…...pS17

Limit of detection……………………………………………………………………………pS18

Structure-based calculations…………………………………………………………………...pS19

Table S2……………………………………………………………………………………..pS20

Job's plot for 2a:c-MYC Pu22 system………………………………………………………pS21

ECD spectra of 2a in the presence of c-MYC Pu22 and c-KIT 2…………………………pS21

Fluorescence displacement assay between 2a and Phen-DC3……………………………pS22

G-tetrad selectivity and PAGE-based competitive studies…………………………………..pS23

Cells stained with 2a and treated with RNase………………………………………………..pS24

Cells stained with 2a and treated with BRACO-19…………………………………………pS25

Fibre analysis…………………………………………………………………………………..pS25

2a in aqueous solution at different temperatures……………………………………………pS26

References……………………………………………………………………………………..pS26

Synthesis

The desired coumarin-3-carboximidamide derivatives (**2a-c**) were synthesized through Knoevenagel condensation of commercially available ortho-hydroxybenzaldehydes by following Scheme S1 as discussed in the main text of the article.

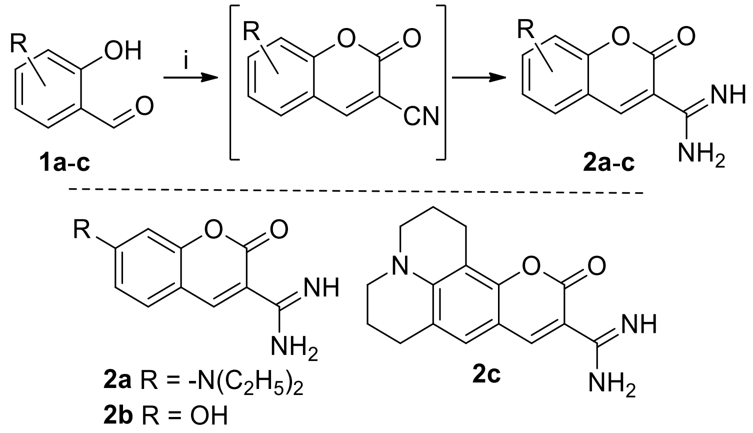

Scheme S1. Reagents and conditions: (i) Ammonium acetate, Ethyl cyanoacetate, absolute ethanol 15min, reflux, (76-87%).

Experimental: All analytical grade reagents and solvents were purchased from Sigma-Aldrich, Fluka and Acros and used as supplied unless stated otherwise. Thin layer chromatography (TLC) was used for monitoring of chemical reactions and performed on aluminum backed silica gel plates (median pore size 60 Å, fluorescent indicator 254 nm) and detected with UV light. Dimethylformamide (DMF) was dried in a solvent drying system (activated molecular sieves in combination with an isocyanate scrubber). ^{1}H and ^{13}C NMR spectra were recorded on Bruker 400 or 600 MHz spectrometers at 298 K and calibrated by using the residual peak of the solvents as the internal standard (DMSO-d_6 : δ H = 2.50 ppm; δ C = 39.50 ppm. The coupling constant values (*J*) are determined in Hertz. The abbreviations used in NMR data are mentioned as, singlet = s, doublet = d, triplet = t, multiplet = m, double doublet = dd, and broad singlet = brs. HRMS was performed by using a Agilent 1290 binary LC System connected to a Agilent 6230 Accurate-Mass TOF LC/MS (ESI+); calibrated with Agilent G1969-85001 ESTOF Reference Mix containing ammonium trifluoroacetate, purine and hexakis (1H, 1H, 3Htetrafluoropropoxy)phosphazine in 90:10 acetonitrile:water.

General procedures for preparation of 3-amidinocoumarin derivatives (2a-c): The reactants ortho- hydroxyl benzaldehydes **1**(**a-c**) (1g, 5.2 mmol), ethyl cyanoacetate (0.58 mL, 5.4 mmol) and ammonium acetate (2.0 g, 26.0 mmol) were taken in a microwave vial with 20 ml of anhydrous ethanol. The sealed vial was then heated at 100 ^{o}C with vigorous stirring. The reaction mixture becomes clear solution within 5 min and reaction was continued stirring at same conditions for

additional 20 min. Soon appearance of precipitates in reaction mixture indicates the formation of product and progress of reaction was monitored on TLC till consumption of starting materials. The precipitates were filtered and washed with saturated bicarbonate solution followed by water and ethanol to give yellow to brown colored residue of desired 3-amidinocoumarin derivatives (**2a-c**) in pure form with 76-87 % yields.

7-(Diethylamino)-2-oxo-2*H*-chromene-3-carboximidamide (2a): The title compound (**2a**) was obtained from the reaction of 4-(diethylamino)-2-hydroxybenzaldehyde (**1a**) with ethyl cyanoacetate as a bright yellow solid in 76% yield by following the general procedure. ^{1}H NMR (400 MHz, DMSO-*d*$_6$) δ (ppm): 8.52 (s, 1H), 7.56 (d, *J* = 8.0 Hz, 1H), 6.77 (dd, *J* = 4.0 & 8.0 Hz, 1H), 6.55-6.57 (m, 4H), 3.47 (q, *J* = 8.0 Hz, 4H), 1.14 (t, *J* = 8.0 Hz, 6H); ^{13}C NMR (150 MHz, DMSO-*d*$_6$) δ (ppm): 161.34, 159.16, 157.13, 152.07, 144.58, 131.21, 112.10, 110.19, 107.97, 96.32, 44.67, 12.79; HRMS: (m/z): calcd for $C_{14}H_{18}N_3O_2$ $(M+H)^+$: 260.1394; obtained 260.1398.

7-Hydroxy-2-oxo-2H-chromene-3-carboximidamide (2b): The title compound (**2b**) was obtained from the reaction of 2,4-dihydroxybenzaldehyde (**1b**) with ethyl cyanoacetate as a bright yellow solid in 87% yield by following the general procedure. ^{1}H NMR (400 MHz, DMSO-*d*$_6$) δ (ppm): 8.02-8.45 (m, 4H), 7.13 (d, *J* = 8.0 Hz, 1H), 6.19 (d, *J* = 8.0 Hz, 1H), 5.85 (s, 1H); ^{13}C NMR (150 MHz, DMSO-*d*$_6$) δ (ppm): 162.15, 161.75, 160.15, 142.51, 132.31, 124.36, 107.40, 104.26, 89.05; HRMS: (m/z): calcd for $C_{10}H_9N_2O_3(M+H)^+$: 205.0608; obtained 205.0613.

11-Oxo-2,3,5,6,7,11-hexahydro-1*H*-pyrano[2,3-f]pyrido[3,2,1-ij]quinoline-10-carboximidamide (2c): The title compound (**2c**) was obtained from the reaction of 9-formyl- 8-hydroxyjulolidine (**1c**) with ethyl cyanoacetate as a brown-red solid in 77% yield by following the general procedure. ^{1}H NMR (400 MHz, DMSO-*d*$_6$) δ (ppm): 8.48 (s, 1H), 7.91(brs, 3H), 7.12 (s, 1H), 3.32-6.3.36 (m, 4H), 2.71-2.75 (m, 4H), 1.86-1.91 (m, 4H); ^{13}C NMR (150 MHz, DMSO-*d*$_6$) δ (ppm): 161.04, 160.09, 152.33, 148.50, 145.74, 127.29, 120.02, 107.64, 105.18, 50.04, 49.52, 27.22, 21.00, 20.05, 19.98; HRMS: (m/z): calcd for $C_{16}H_{18}N_3O_2$ $(M+H)^+$: 284.1394; obtained 284.1397.

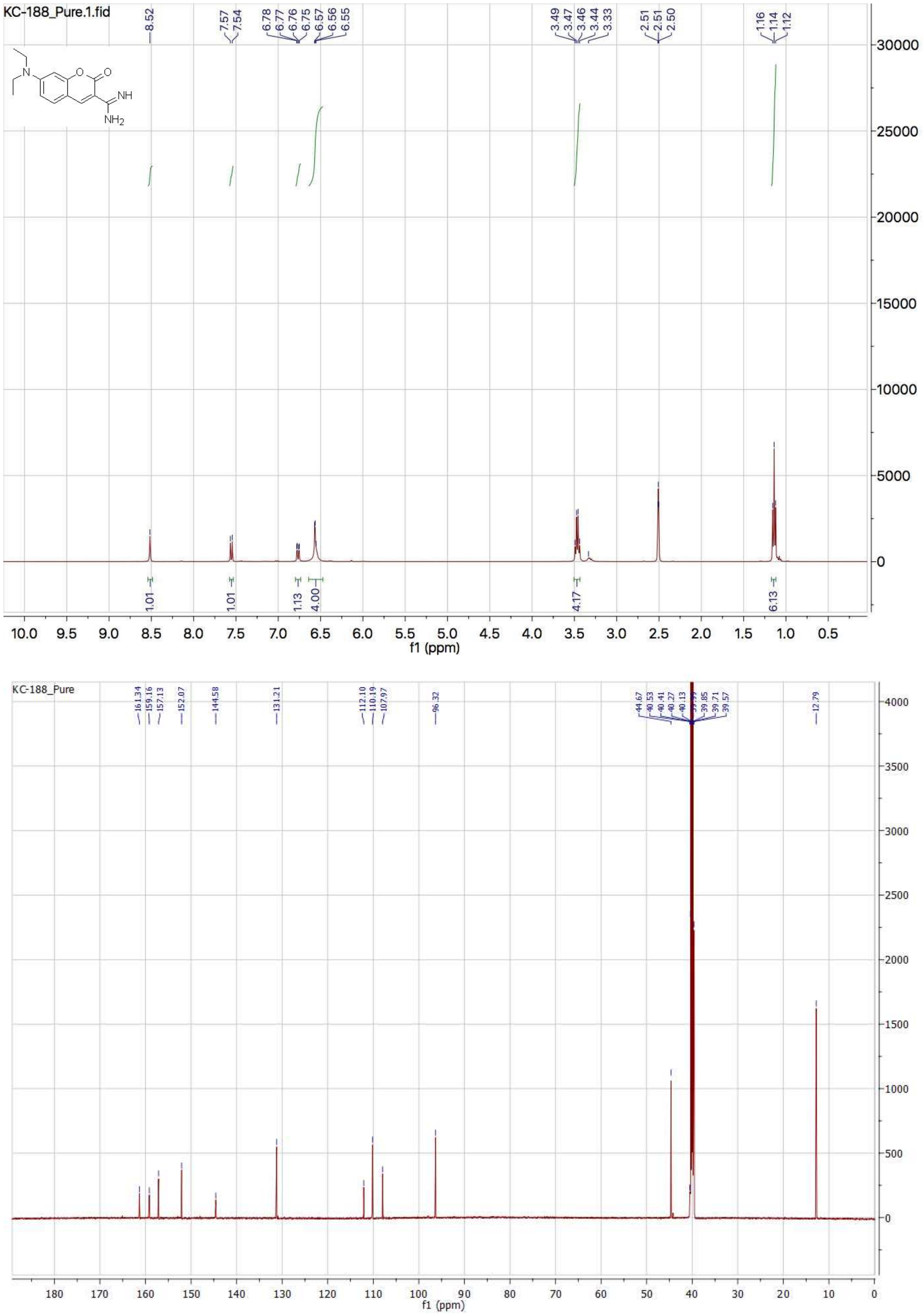

Figure S1. ^{1}H and ^{13}C NMR spectra of 7-(diethylamino)-2-oxo-2*H*-chromene-3-carboximidamide (**2a**).

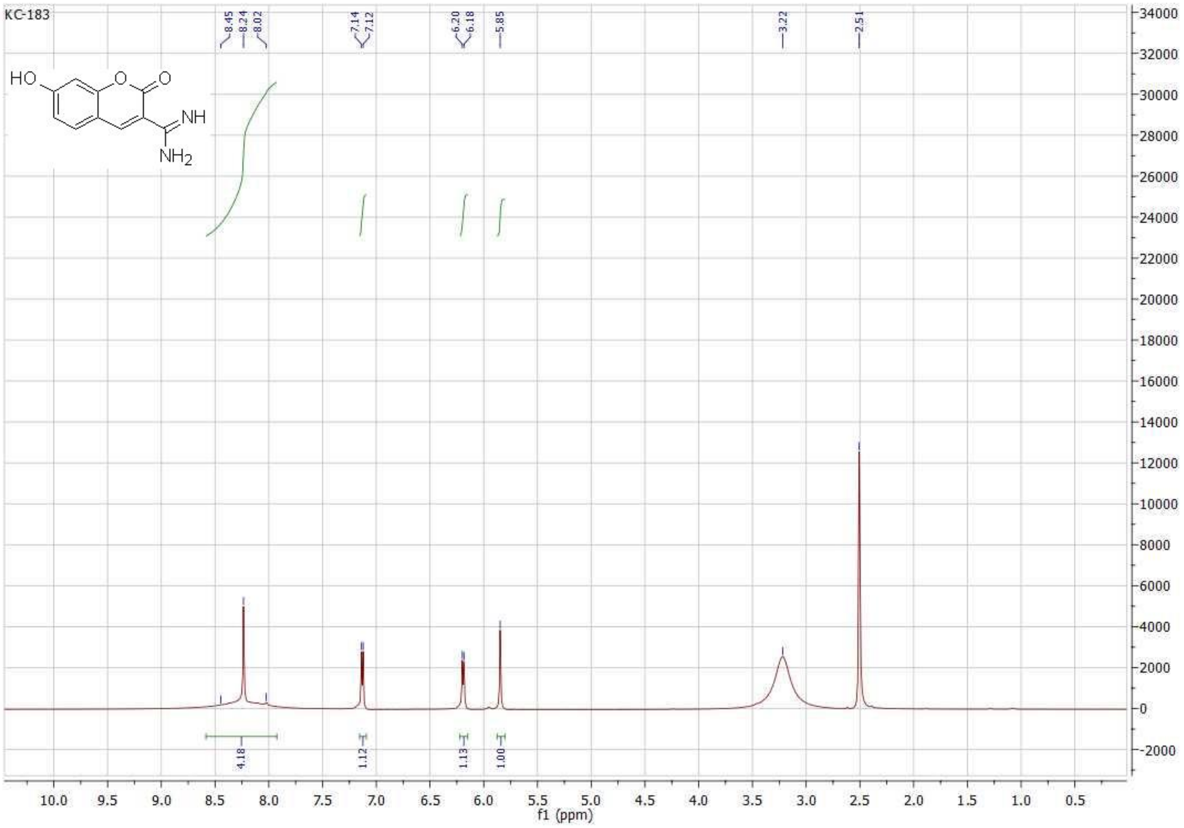

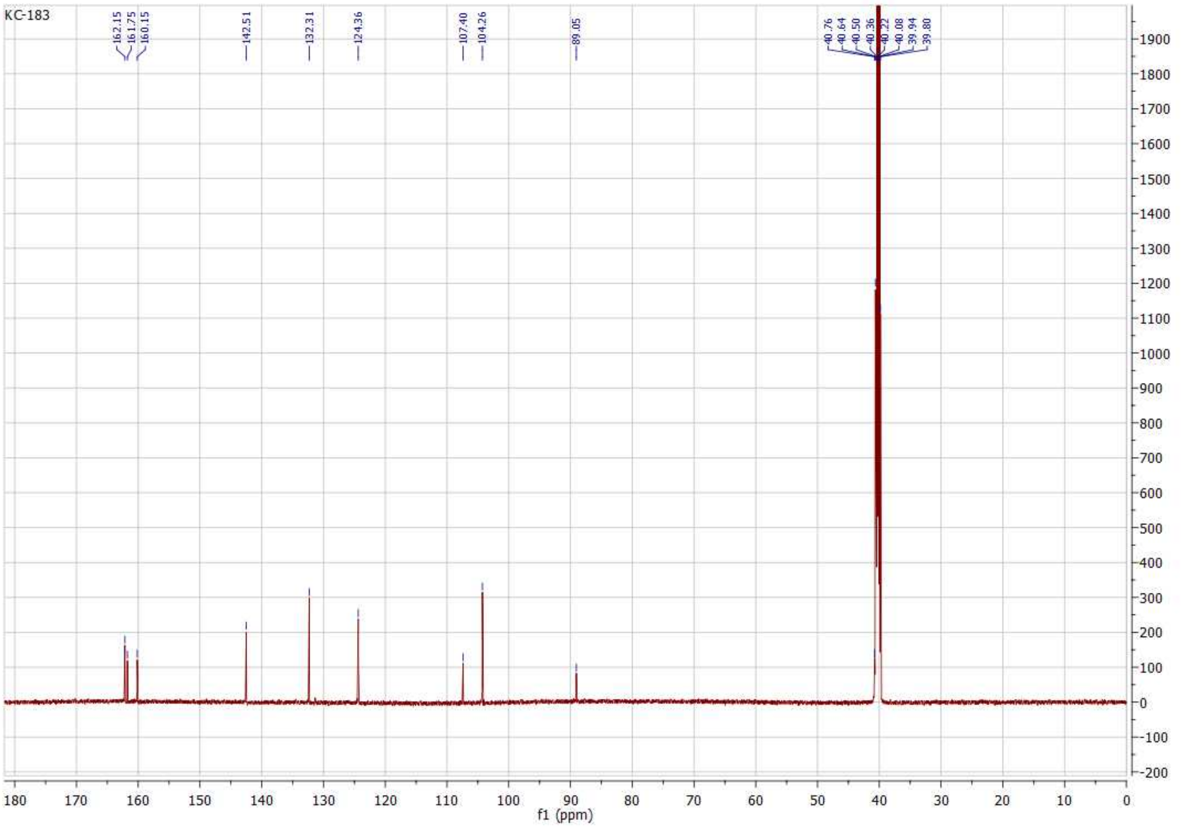

Figure S2. ^{1}H and ^{13}C NMR spectra of 7-hydroxy-2-oxo-2*H*-chromene-3-carboximidamide (**2b**).

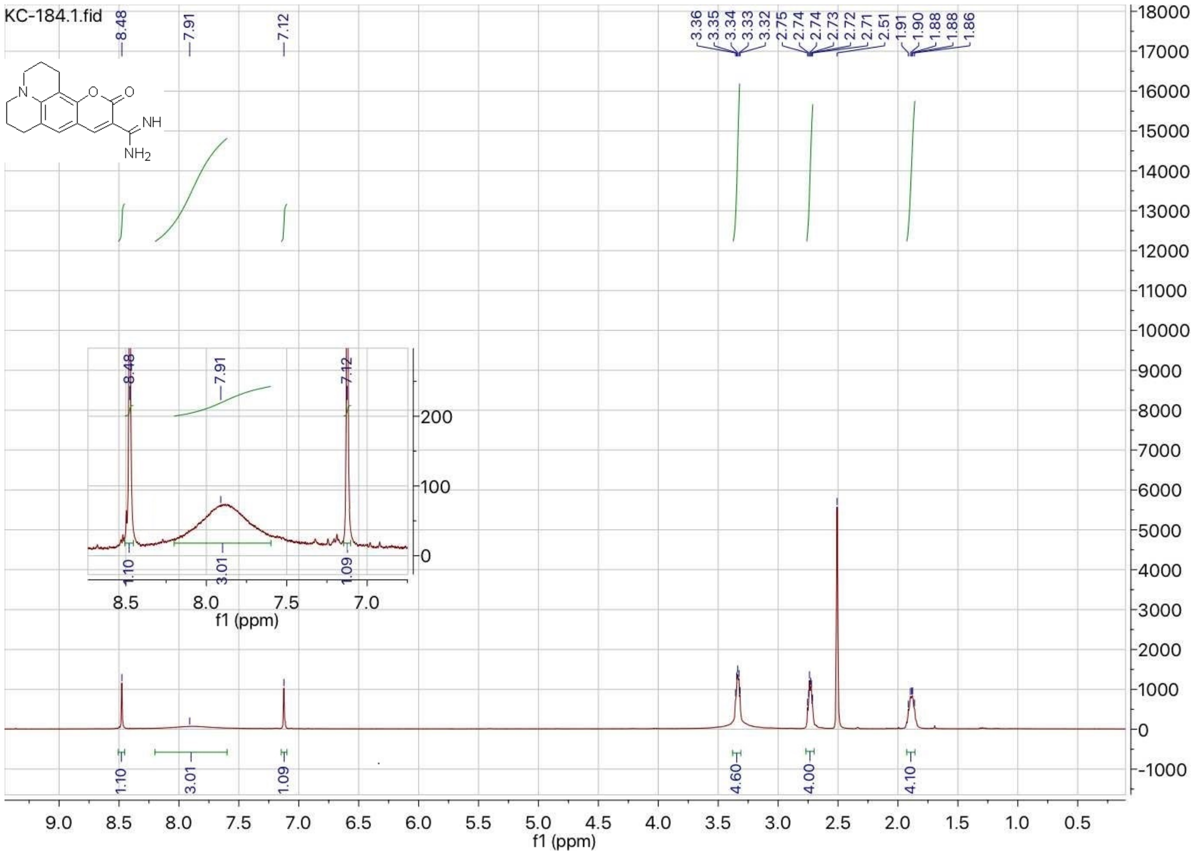

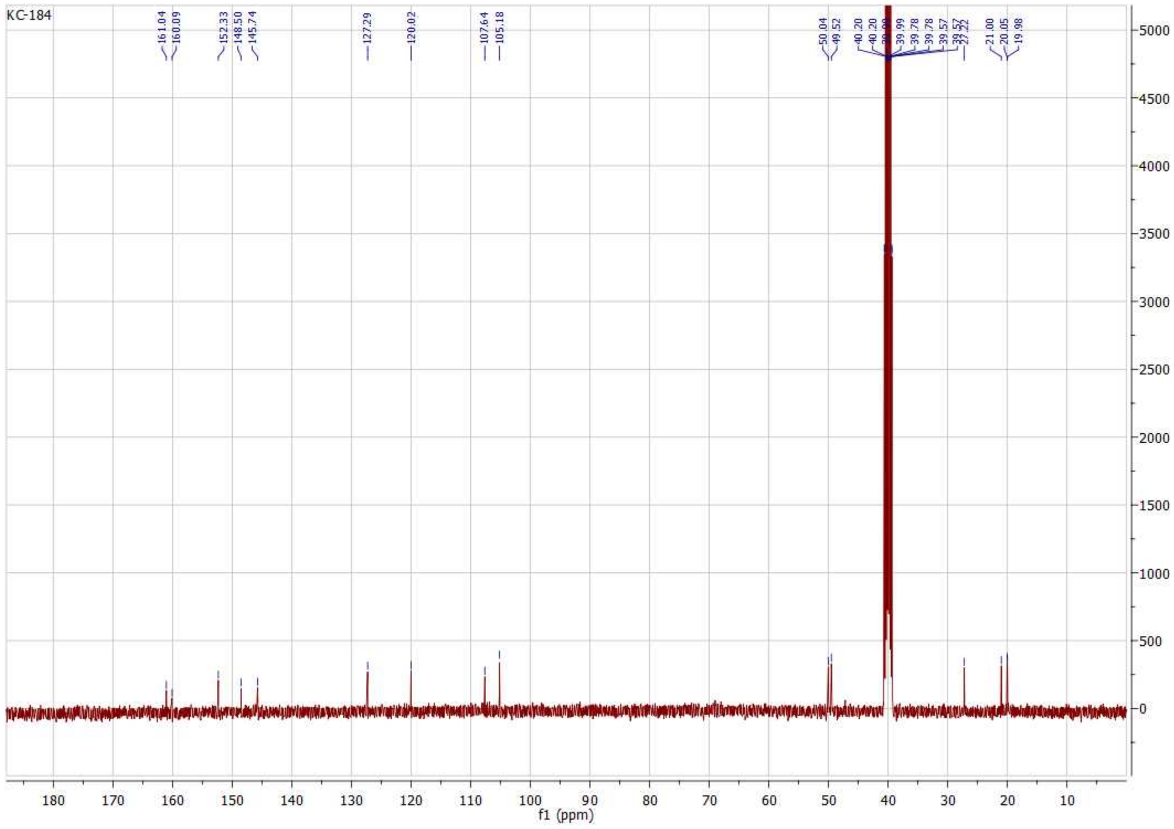

Figure S3. ^{1}H and ^{13}C NMR spectra of 11-Oxo-2,3,5,6,7,11-hexahydro-1*H*-pyrano[2,3-f]pyrido[3,2,1-ij]quinoline-10- carboximidamide (**2c**)

Solvent-dependent optical studies

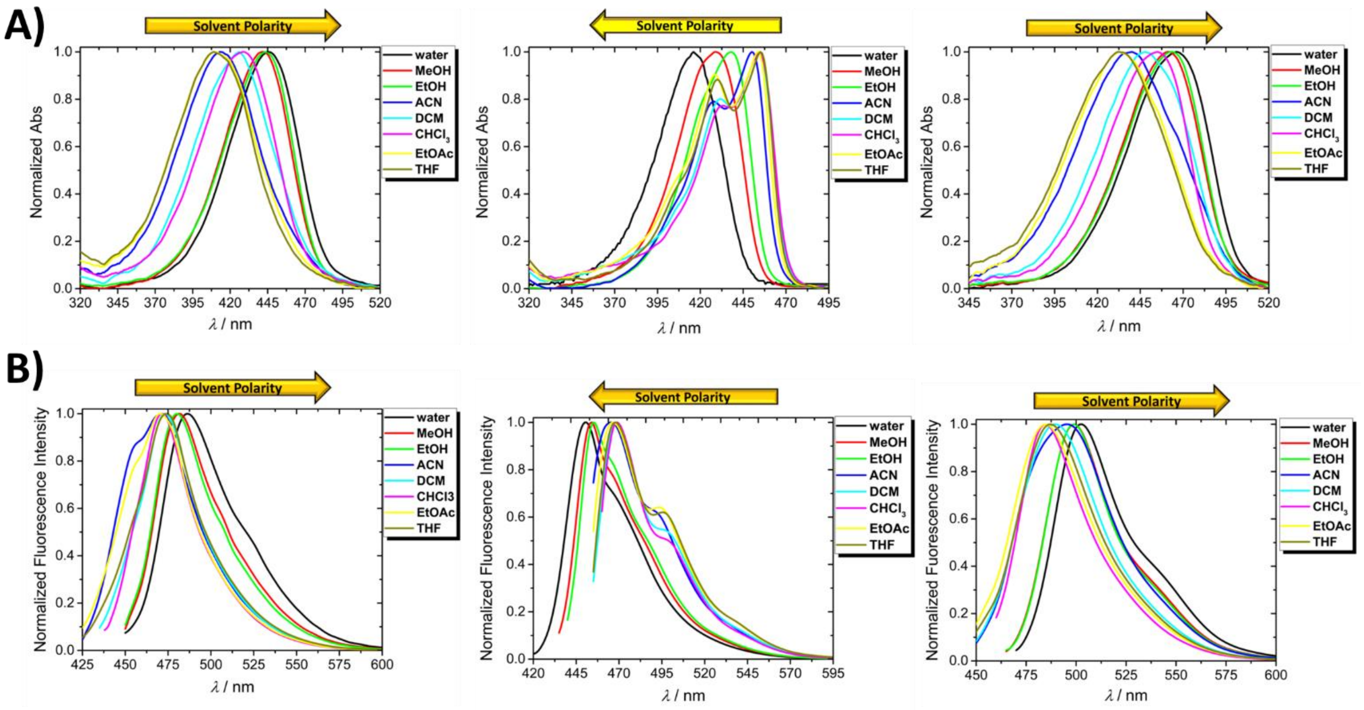

Figure S4. A) Solvent-dependent UV/Vis spectra of **2a** (left panel), **2b** (central panel) and **2c** (right panel). Compound concentration = 3.0 μM. **B)** Solvent-dependent emission spectra of **2a** (left panel), **2b** (central panel) and **2c** (right panel). Compound concentration **=** 3.0 μM ($\lambda_{ex} = \lambda_{max}$).

Spectrophotometric and fluorimetric titration of 2b in the presence of G4 structures

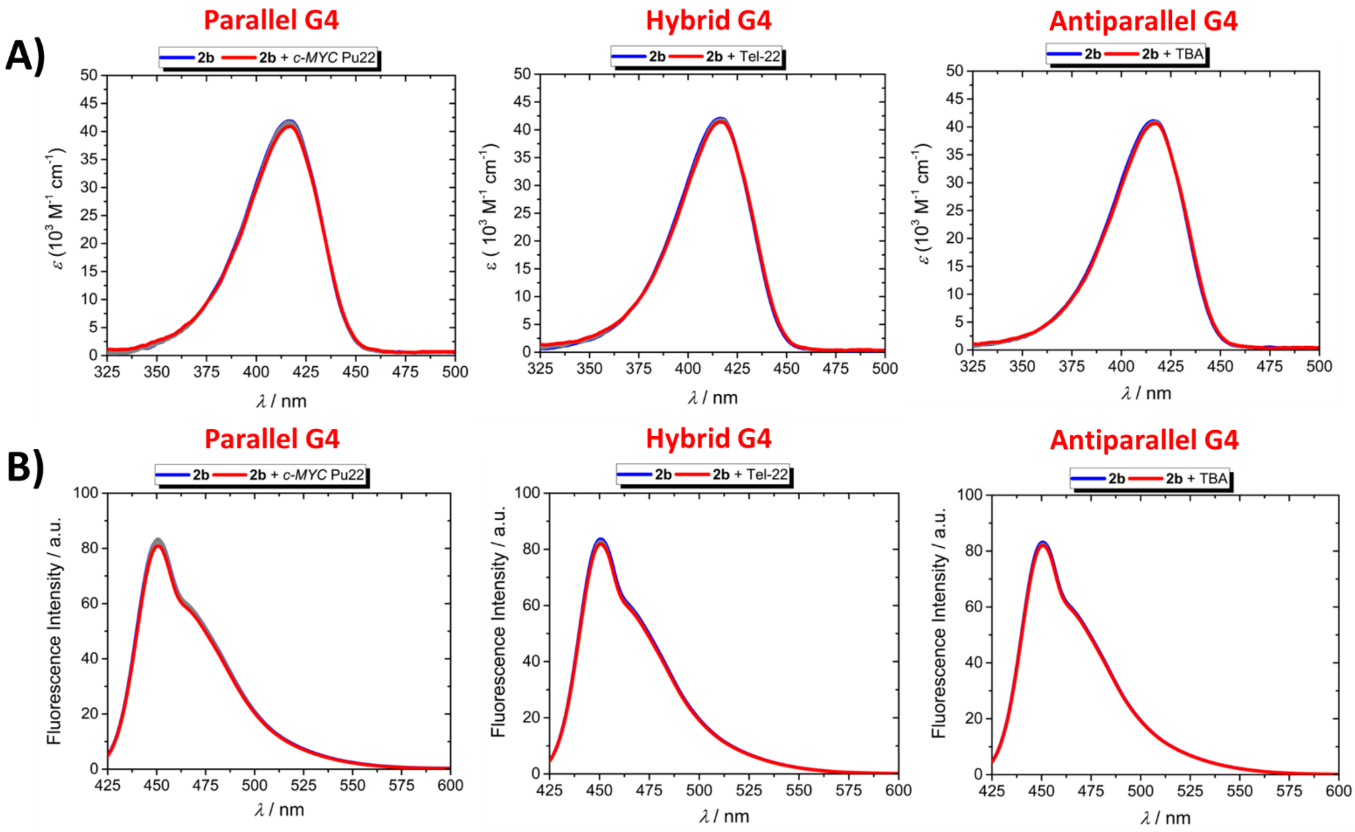

Figure S5. A) Spectrophotometric titration of a buffered **2b** solution (3.0 μM, C_{KCl} = 100 mM, Tris buffer 50.0 mM, pH 7.5) upon addition of *c-MYC* Pu22 (left panel), Tel-22 (central panel) or TBA (right panel) at 25 °C (blue and red lines correspond to the spectra at 0.0 and 3.0 eq., respectively). **B)** Fluorimetric titration of a buffered **2b** solution (1.5 μM, C_{KCl} = 100 mM, Tris buffer 50.0 mM, pH 7.5) upon addition of *c-MYC* Pu22 (left panel), Tel-22 (central panel) or TBA (right panel) at 25 °C (blue and red lines correspond to the spectra at 0.0 and 5.0 eq., respectively).

G4 characterization

Table S1. List of all oligonucleotides used in this study for spectroscopic and PAGE studies.

Name	Sequence 5´-3´	Length (bp)	Molar extinction coefficient ($M^{-1}cm^{-1}$)[a]	GC%	Topology and description
***c-MYC* Pu22**	TGAGGGTGGGTAGGGTGGGTAA	22	228700	59.1	parallel
***c-MYC* Pu24T**	TGAGGGTGGTGAGGGTGGGGAAGG	24	248200	66.7	parallel
***c-MYC* sG4**	GGGTGGGTAGGGTGGG	16	162700	75	parallel
***BCL*-2**	GGGCGCGGGAGGGAATTGGCGGGG	24	237400	79.2	parallel
**VAV-1**	GGGCAGGGAGGGAACTGGG	19	194700	73.7	parallel
***c-KIT* 2**	CCCGGGCGGGCGCGAGGGAGGGGAGG	26	253400	88.5	parallel (snap-back)
**Tel-22**	AGGGTTAGGGTTAGGGTTAGGG	22	228500	54.5	hybrid/anti-parallel
**Bom17**	GGTTAGGTTAGGTTAGG	17	174600	47.1	anti-parallel
**TBA**	GGTTGGTGTGGTTGG	15	143300	60	anti-parallel
**sC4**	CCCACCCTACCCACCC	16	132500	75	single stranded
***ss*-DNA**	GGATGTGAGTGTGAGTGTGAGG	22	227000	54.5	single stranded
**Genomic *ds*-DNA[b]**	/	~2000	13200	/	double stranded
**GC-rich *ds*-DNA[c]**	TGAGGGTGGGTAGGGTGGGTAA<br>TTACCCACCCTACCCACCCTCA	22	/	59.1	double stranded
**s.c. *ds*-DNA[d]**	CAATCGGATCGAATTCGATCCGATTG	26	253200	46.2	double stranded

[a] The molar extinction coefficient was calculated using the oligo analyser tool on the IDT web site. [b] Salmon sperm DNA. [c] GC-rich *ds*-DNA folding was induced by mixing an equimolar concentration of both strands and heating the sample at 95°C for 5 min in the presence of 100 mM KCl and then slowly allowed to reach room temperature overnight. [d] Self-complementary (s.c.) *ds*-DNA.

Evidences for G4 folding sequences, used in this study, were provided by electronic circular dichroism measurements and isothermal difference spectra (IDS) defined as the difference between unfolded and prefolded UV/Vis spectra. ECD spectra were normalized to $\Delta\varepsilon$ ($M^{-1}\cdot cm^{-1}$) = θ / (32980 $\cdot$ c $\cdot$ l) based on G4-strand concentration, where θ is the ECD ellipticity in millidegrees, c is DNA concentration in mol/L, and l is the path length in cm.[1, 2] Representative examples of ECD and IDS spectra for parallel, hybrid and antiparallel G4 morphologies is provided in Figures S6-S8.

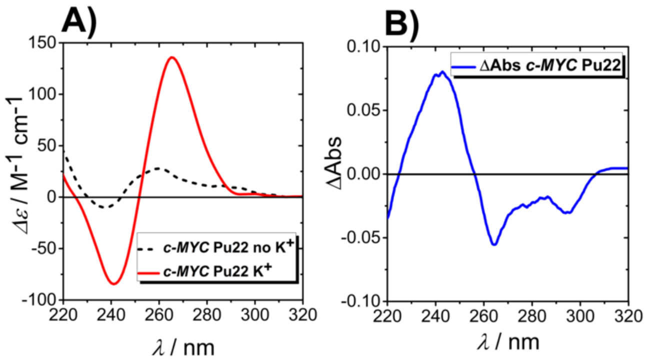

Figure S6. A) ECD spectra of *c-MYC* Pu22 in the absence (black dashed line) and presence of K^+ (red line). **B)** IDS of *c-MYC* Pu22 (blue line). All the measurements were performed in Tris buffer 10.0 mM.

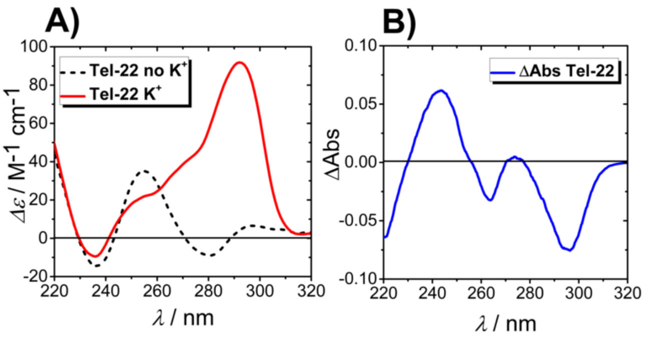

Figure S7. A) ECD spectra of Tel-22 in the absence (black dashed line) and presence of K^+ (red line). **B)** IDS of Tel-22 (blue line). All the measurements were performed in Tris buffer 10.0 mM.

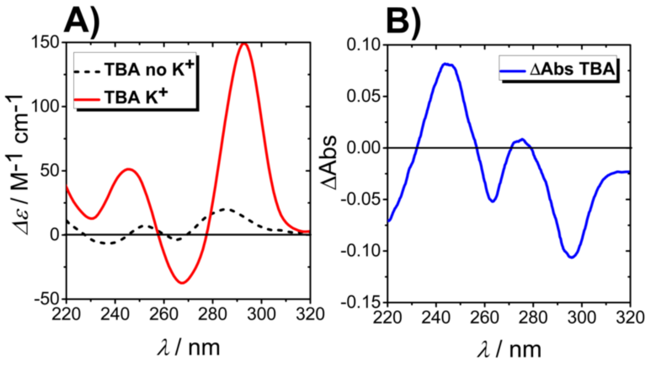

Figure S8. A) ECD spectra of TBA in the absence (black dashed line) and presence of K^+ (red line). **B)** IDS of TBA (blue line). All the measurements were performed in Tris buffer 10.0 mM.

Binding of 2a to various G4 and non-G4 structures

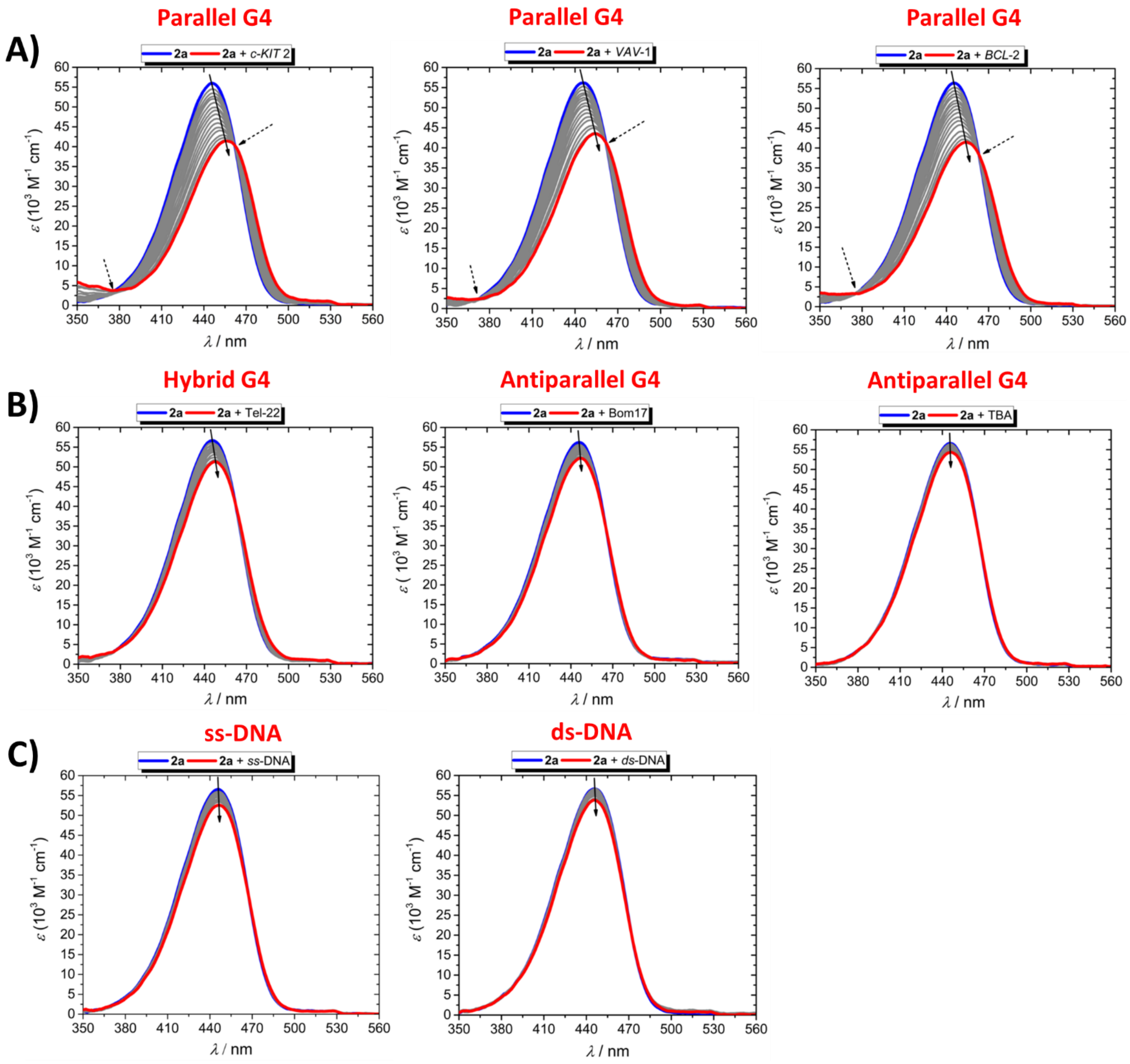

Figure S9. Spectrophotometric titration of a buffered **2a** solution (3.0 µM, C_{KCl} = 100 mM, Tris buffer 50.0 mM, pH 7.5) upon addition of **A)** parallel G4s, **B)** hybrid and antiparallel G4s or **C)** non-G4 structures at 25 °C (blue and red lines correspond to the spectra at 0.0 and 11.33 eq., respectively). The solid and dashed arrows show the evolution of the binding profile and the appearance of isosbestic points, respectively.

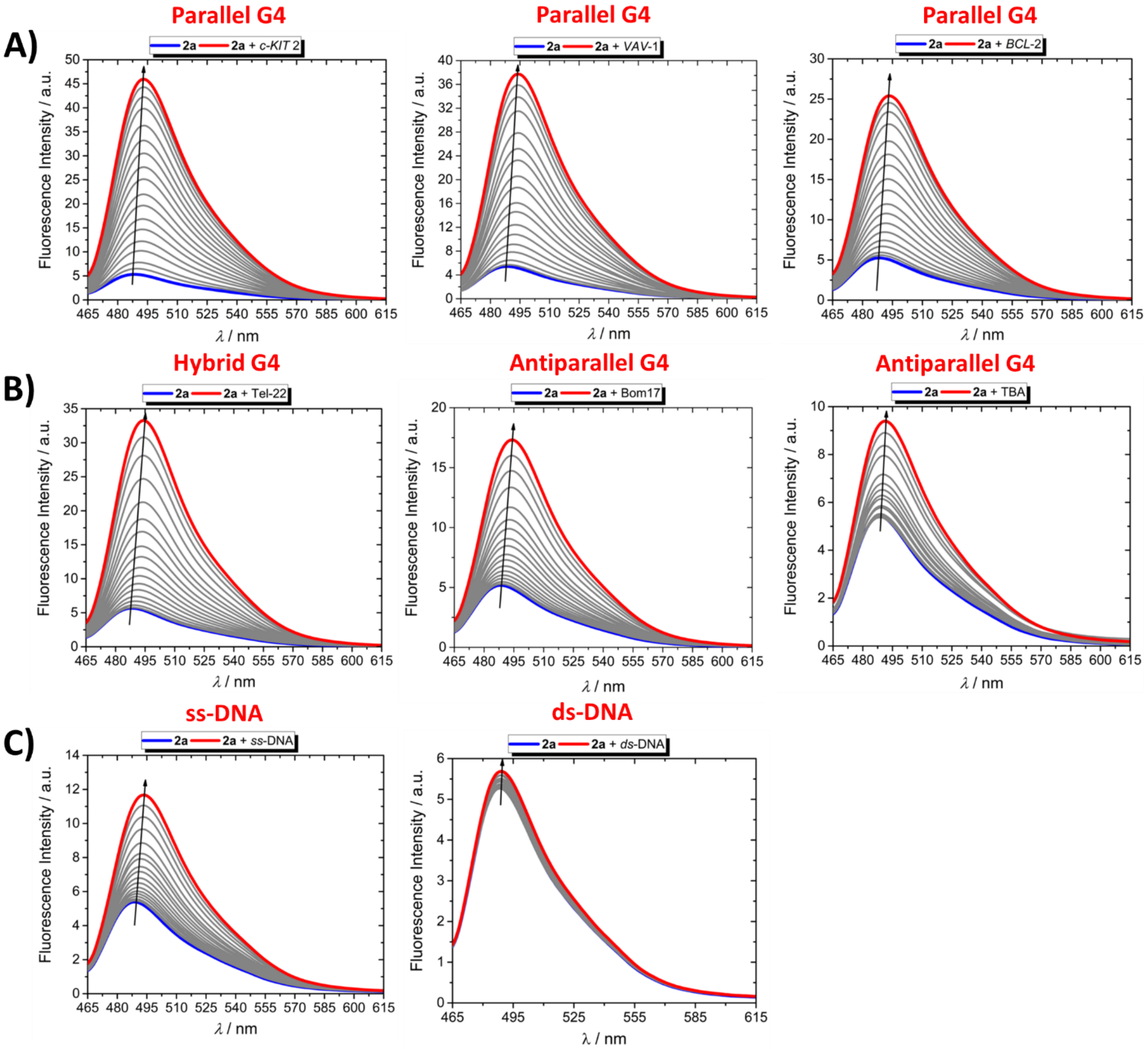

Figure S10. Fluorimetric titration of a buffered **2a** solution (1.5 µM, C_{KCl} = 100 mM, Tris buffer 50.0 mM, pH 7.5) upon addition of **A)** parallel G4s, **B)** hybrid and antiparallel G4s or **C)** non-G4 structures at 25 °C (blue and red lines correspond to the spectra at 0.0 and 23.33 eq., respectively). The arrow shows the evolution of the binding profile.

Binding of 2c to various G4 and non-G4 structures

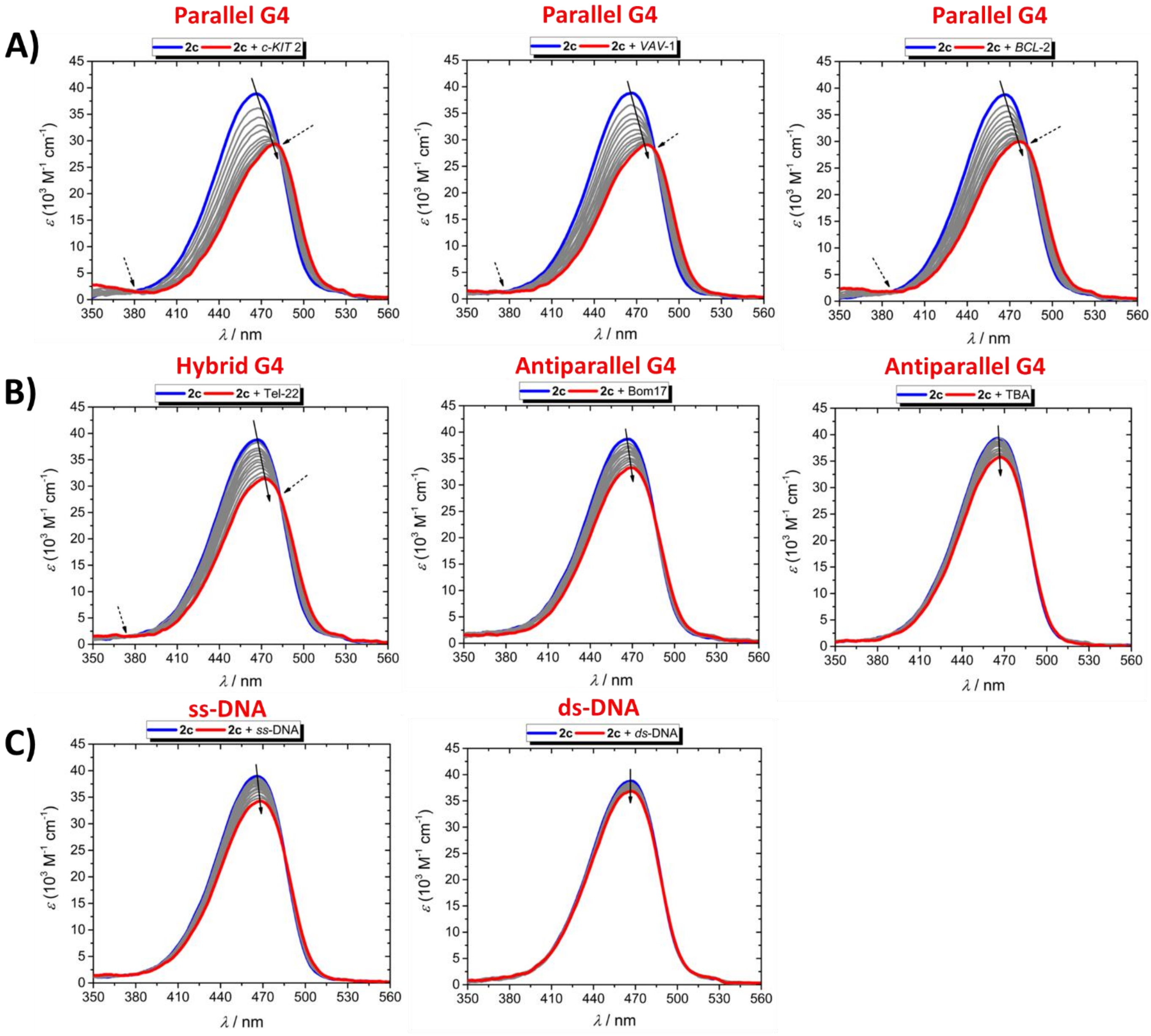

Figure S11. Spectrophotometric titration of a buffered **2c** solution (3.0 μM, C_{KCl} = 100 mM, Tris buffer 50.0 mM, pH 7.5) upon addition of **A)** parallel G4s, **B)** hybrid and antiparallel G4s or **C)** non-G4 structures at 25 °C (blue and red lines correspond to the spectra at 0.0 and 4.66 eq., respectively). The solid and dashed arrows show the evolution of the binding profile and the appearance of isosbestic points, respectively.

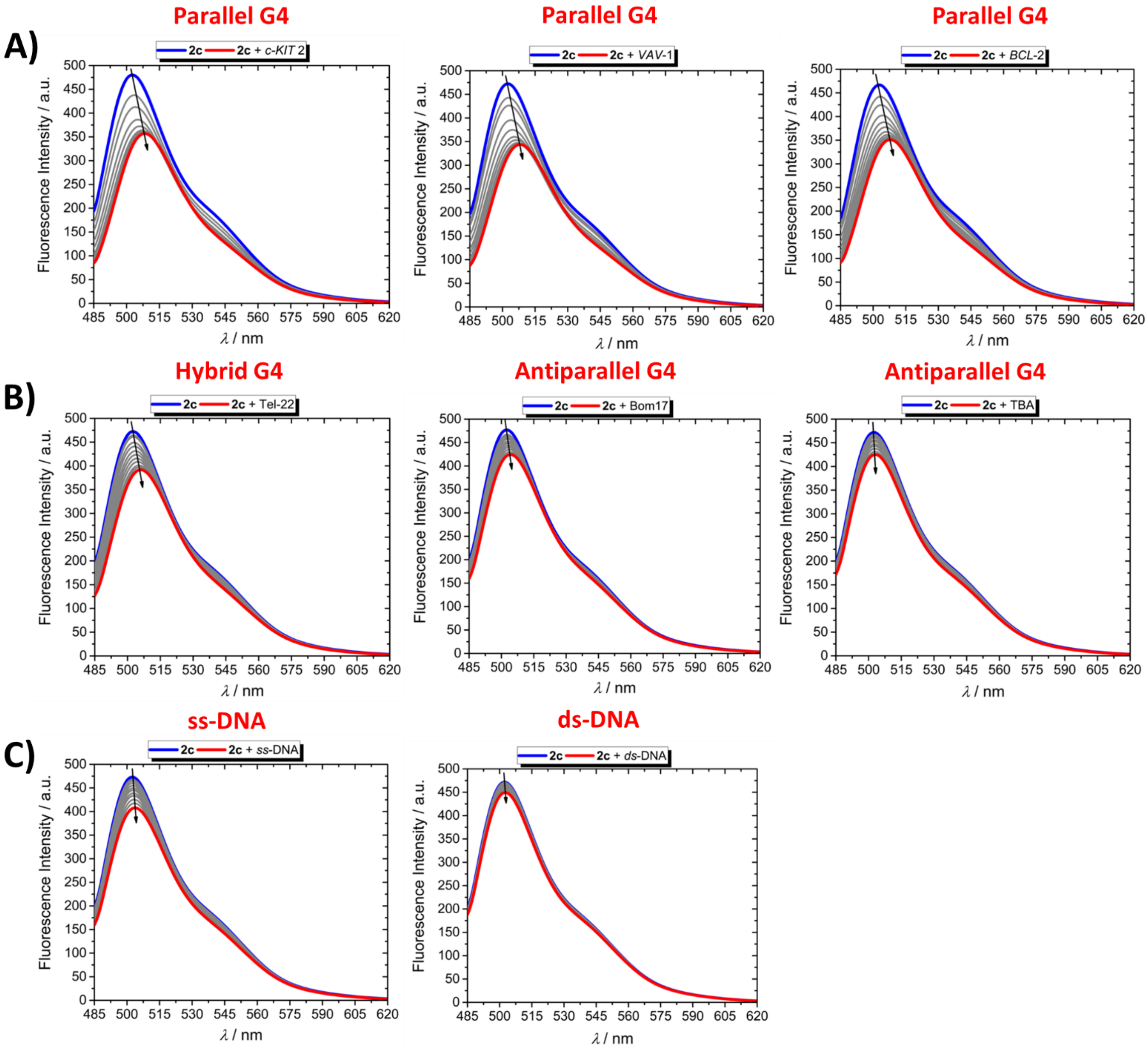

Figure S12. Fluorimetric titration of a buffered **2c** solution (1.5 µM, C_{KCl} = 100 mM, Tris buffer 50.0 mM, pH 7.5) upon addition of **A)** parallel G4s, **B)** hybrid and antiparallel G4s or **C)** non-G4 structures at 25 °C (blue and red lines correspond to the spectra at 0.0 and 6.66 eq., respectively). The arrow shows the evolution of the binding profile.

Parallel G4 recognition mediated by 2a

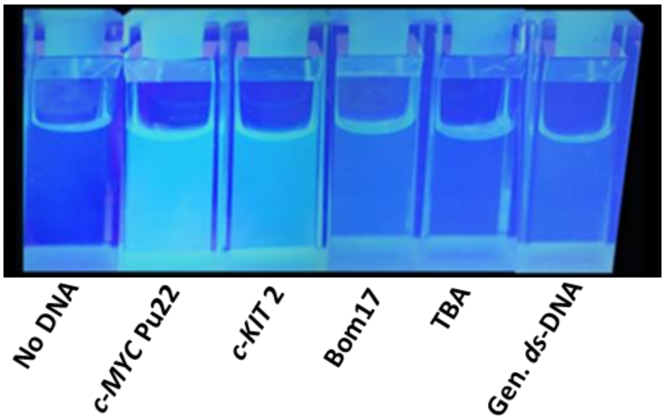

Figure S13. Selective parallel G4 discrimination of **2a**. Different DNA oligonucleotides (20 µM) were incubated with **2a** (5 µM). Illumination was from a UV lamp operating at 312 nm.

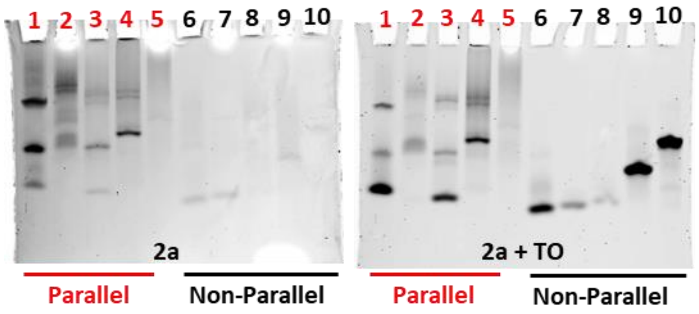

Figure S14. Non-denaturing PAGE images of mono-, and multimeric G4 structures and non-G4 structures stained with **2a** (left-panel). The same gel was also co-stained with TO (right panel) to show the specificity of binding. The reactions included 5 µM **2a**, 5 µM TO, and 25 µM oligo (*c-MYC* Pu22 (lane 1), *c-MYC* sG4 (lane 2), *c-MYC* Pu24T (lane 3), *c-KIT* 2 (lane 4), *VAV*-1 (lane 5), Tel-22 (lane 6), Bom17 (lane 7), TBA (lane 8), GC-rich *ds*-DNA (lane 9), and self-complementary *ds*-DNA (lane 10), λ_{exc} = 457 nm.

Enhancement of the fluorescence intensity of 2a in viscous buffer supports the TICT process

TICT is an electron transfer process rather common in molecules that feature electron donating and electron accepting groups linked through a single bond.[3] Since the relaxation pathway can be tuned by changing the nature of the substituents, local polarity and steric restrictions, TICT-based fluorophores hold great promise in biomedical imaging and diagnostics.[3]

Therefore, we determined whether the fluorescence enhancement supports a TICT process driven by the restriction of the intramolecular rotation of the probe's excited state and consequently activating the radiative channels. To restrict the intramolecular rotation of **2a**, we performed titration experiments in different percentages of glycerol aiming to gradually enhance the viscosity of the medium and thereby gradually restrict the intramolecular rotation of **2a**.[4] Indeed, upon increasing the glycerol percentage, a strong emission enhancement of **2a** was observed (~18-fold enhancement between 0% and 100% glycerol) (Figure S15). Therefore, these viscosity-dependent fluorescence changes clearly support a TICT process due to the restriction of the intramolecular rotation of the **2a**'s diethyl-amino group.

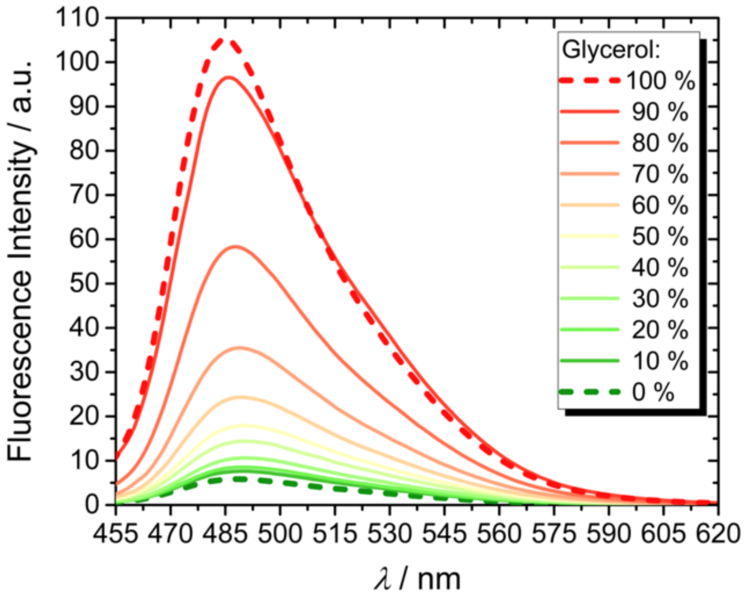

Figure S15. Viscosity-dependent emission spectra of **2a** (1.5 µM; λ_{exc} = 445 nm). **2a** shows enhanced fluorescence intensity with increasing glycerol content.

Limit of detection

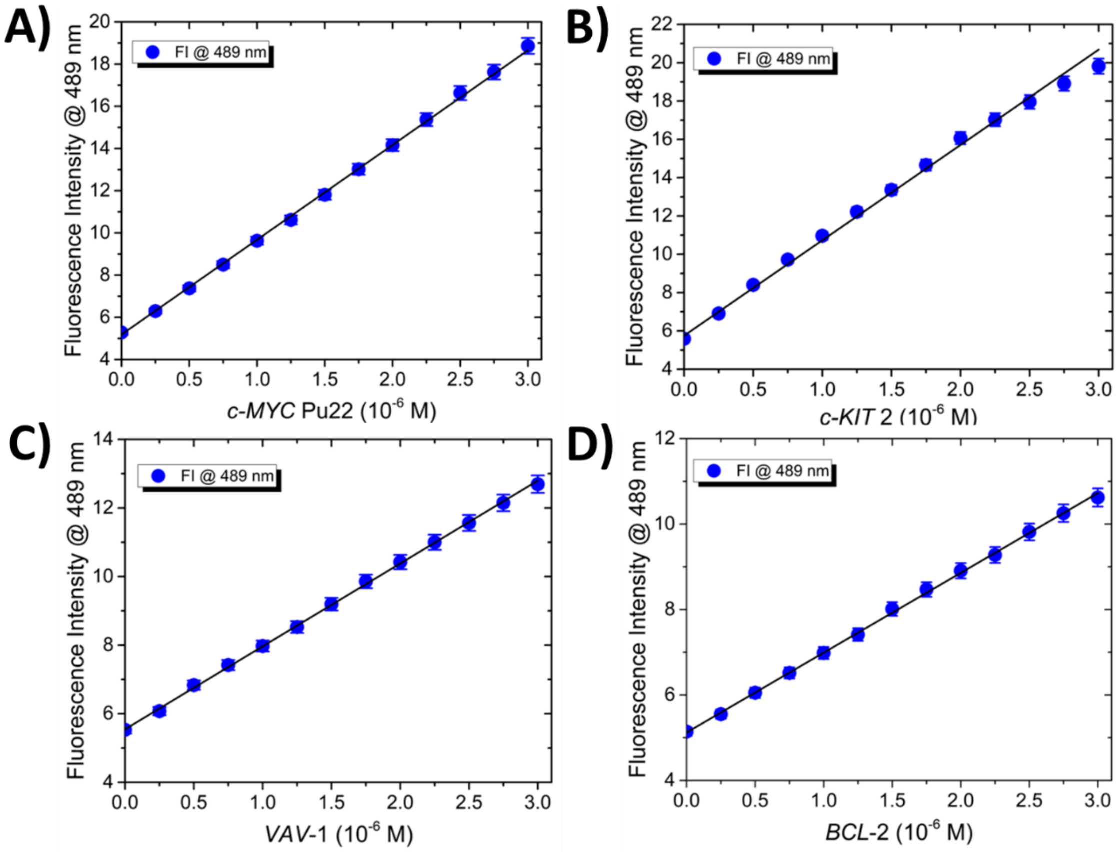

Figure S16. Calculated limit of detection (LOD) for **A) 2a**-*c*-*MYC* Pu22, **B) 2a**-*c*-*KIT* 2, **C) 2a**-*VAV*-1 and **D) 2a**-*BCL*-2 (KCl = 100 mM, Tris buffer c = 50 mM, pH = 7.5, λ_{exc} = 461 nm).

Structure-based calculations

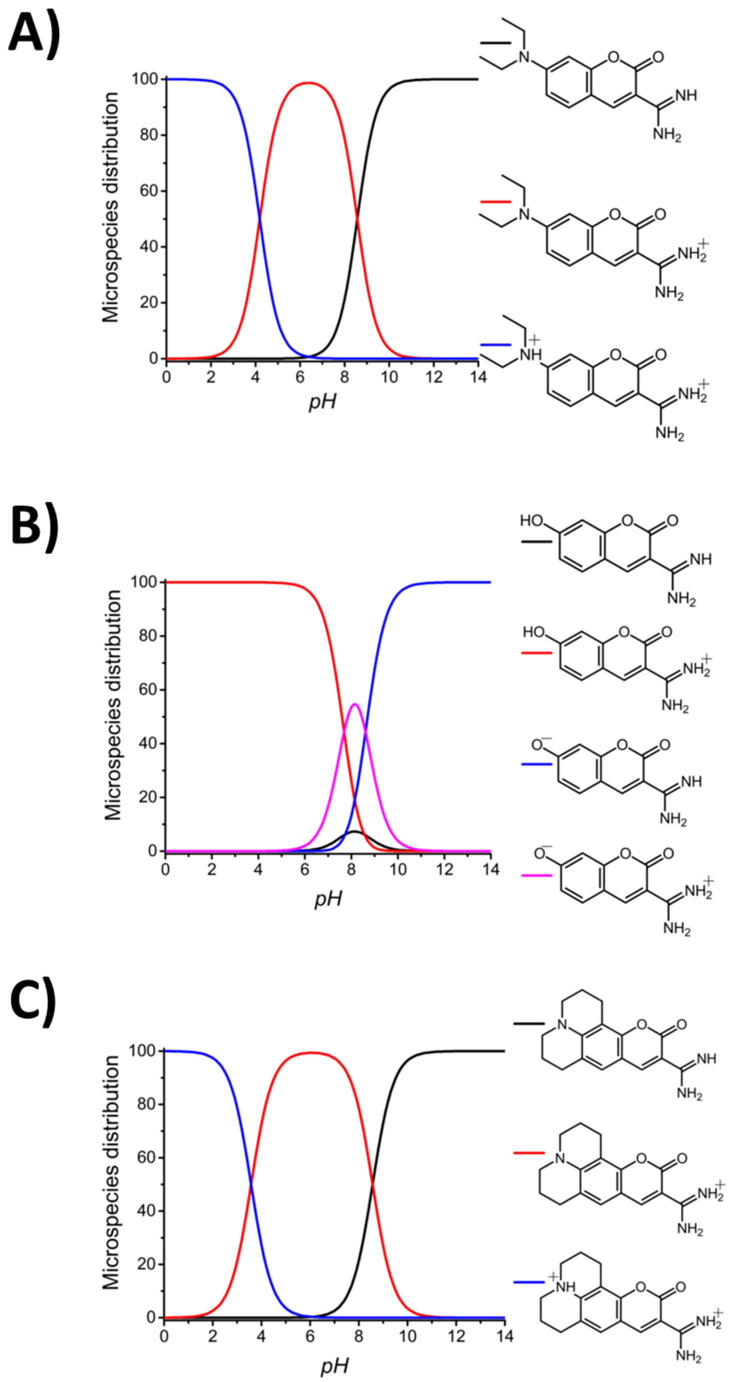

Figure S17. Structure-based calculations of the microspecies distribution of **A) 2a**, **B) 2b** and **C) 2c** predicted by using MarvinSketch software.

Table S2. Dissociation constant for **2a** and **2c** compounds in the presence of G4 structures.

Compound	G4 Topology	K_d / μM[a]	$Cov_{(Fit)}$	Error (%)	K_d / μM[b]	$Cov_{(Fit)}$	Error (%)
**2a**							
c-MYC Pu22	parallel	13.0	0.0005	0.19	6.7	0.0009	0.42
c-KIT 2	parallel	9.1	0.0004	0.20	6.7	0.0008	0.37
VAV-1	parallel	15.0	0.0002	0.13	10.1	0.0016	0.46
BCL-2	parallel	14.6	0.0004	0.17	10.9	0.0018	0.48
Tel-22	hybrid	45.2	0.0002	0.08	N.D.	N.D.	N.D.
**2c**							
c-MYC Pu22	parallel	0.3	0.0033	2.7	0.4	0.0040	2.78
c-KIT 2	parallel	0.7	0.0018	1.6	0.3	0.0039	3.09
VAV-1	parallel	0.3	0.0010	4.64	0.8	0.0037	1.84
BCL-2	parallel	0.5	0.0073	3.17	1.1	0.0034	1.57
Tel-22	hybrid	3.8	0.0034	0.86	5.3	0.0021	0.58

[a] Fitting with 1:1 binding model was obtained with Bindfit by using multiple global fitting methods (Nelder–Mead method) on the fluorimetric data. The covariance fitting ($Cov_{(fit)}$) and the error in % are also reported to show the goodness of fit. [b] Fitting with 1:1 binding model was obtained with Bindfit by using multiple global fitting methods (Nelder–Mead method) on the spectrophotometric data. The covariance fitting ($Cov_{(fit)}$) and the error in % are also reported to show the goodness of fit.

Job's plot for 2a:*c-MYC* Pu22 system

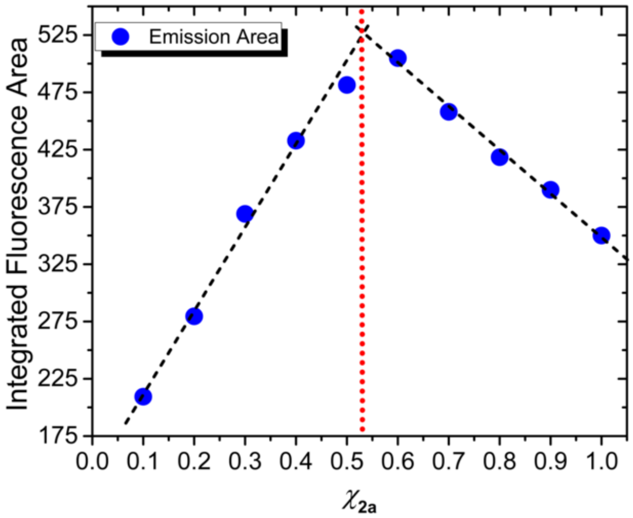

Figure S18. Job's plot method for assessing the **2a**:*c–MYC* Pu22 stoichiometry (KCl = 100 mM, Tris buffer c = 50 mM, pH = 7.5, λ_{exc} = 461 nm). The vertical red dotted line shows the stoichiometry of the system.

ECD spectra of 2a in the presence of *c-MYC* Pu22 and *c-KIT* 2

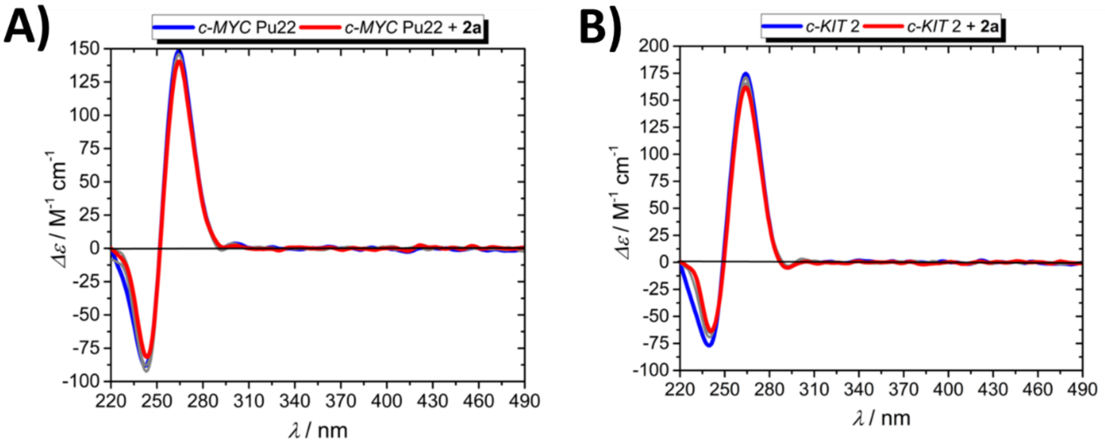

Figure S19. ECD spectra of **A)** *c-MYC* Pu22 and **B)** *c-KIT* 2upon addition of **2a** (blue and red lines correspond to the spectra at 0.0 eq. and 2.0 eq., respectively). Experimental conditions: Tris buffer 50.0 mM, pH 7.5, KCl 100 mM, 25 °C.

Fluorescence displacement assay between 2a and Phen-DC$_3$

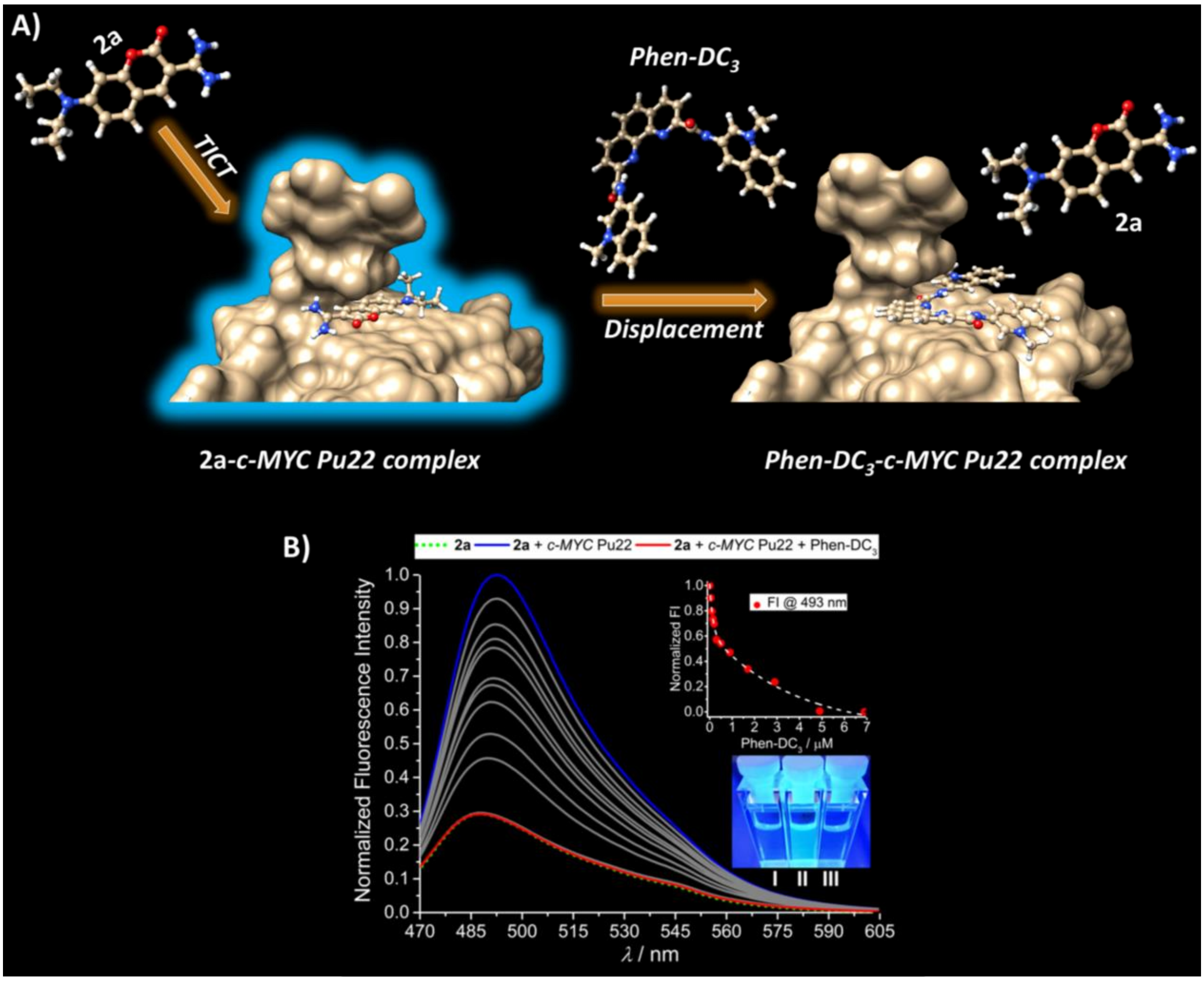

Figure S20. A) Schematic representation of the **2a**-*c-MYC* Pu22-Phen-DC$_3$ displacement assay. **B)** The direct competition for the same binding sites with the well-known G4 end-stacker Phen-DC$_3$ results in the displacement of **2a** from the G4 template with the consequent turning off of the fluorescent response. The Phen-DC$_3$ displacement assay included: 0.5 µM **2a**, 2.5 µM *c-MYC* Pu22, 0–6.9 µM Phen-DC$_3$, 100 mM KCl, and 50 mM Tris buffer (pH 7.5), λ_{exc} = 461 nm. Insets: the extent of Phen-DC$_3$-driven **2a** displacement monitored at 493 nm (top-right panel) and the associated optical changes (bottom-right panel). **2a** is non-emissive in its free state (I). Complexation with *c-MYC* Pu22 induced a clear colour change of the solution (II). Replacement of **2a** on the G4 template by Phen-DC$_3$ caused the fluorescence quenching of **2a** (III).

G-tetrad selectivity and PAGE-based competitive studies

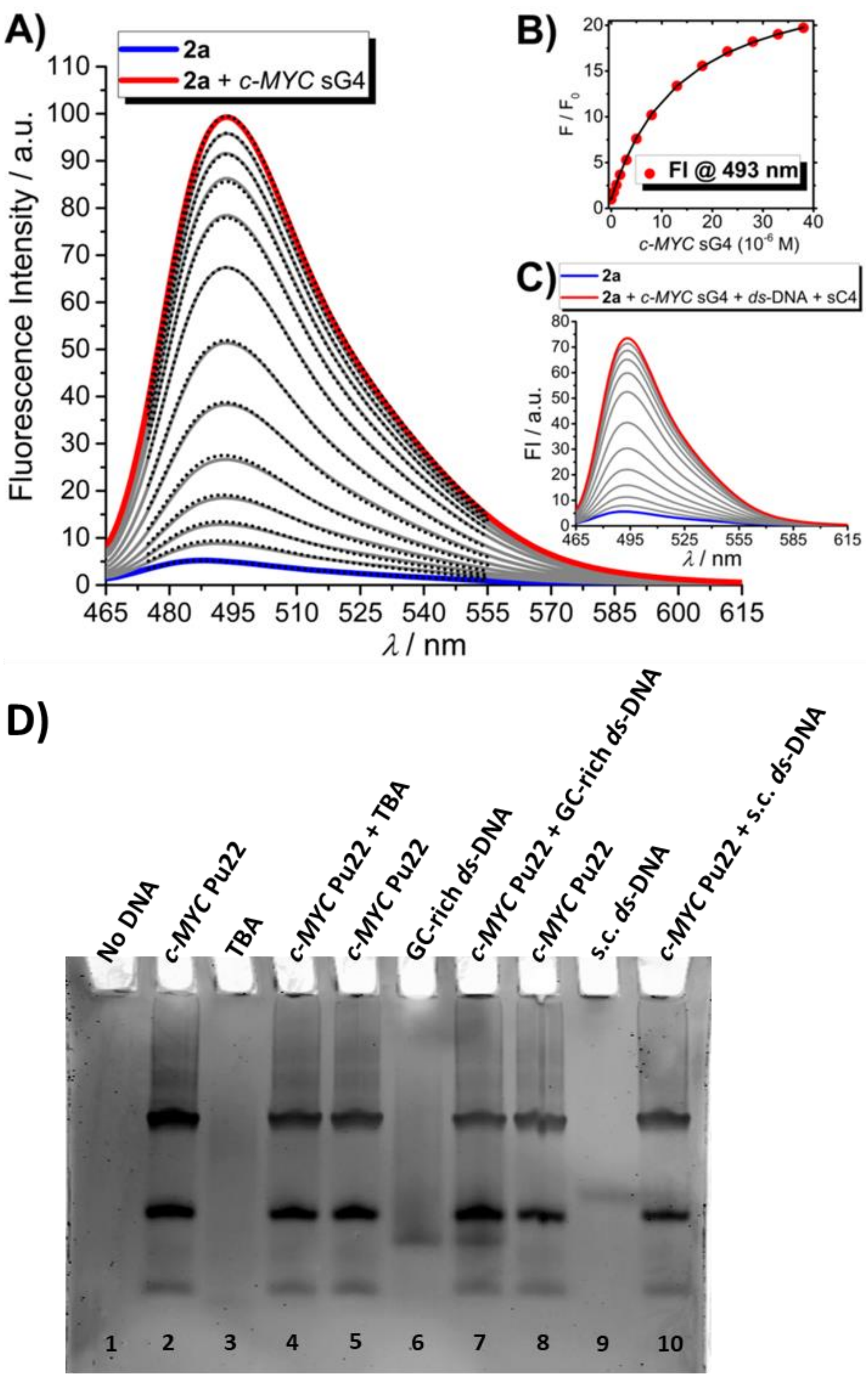

Figure S21. A) Fluorimetric titration of a buffered **2a** solution (1.5 µM **2a**, 100 mM KCl, and 50 mM Tris buffer (pH 7.5)) upon addition of *c-MYC* sG4 at 25°C (blue and red lines correspond to the spectra at 0.0 and 25.3 eq., respectively). The superimposed dotted lines correspond to a 1:1 global fitting model. **B)** The binding isotherm for the **2a**-*c-MYC* sG4 system. **C)** Fluorimetric titration of a buffered **2a** solution (1.5 µM **2a**, 100 mM KCl, and 50 mM Tris buffer (pH 7.5)) upon addition of *c-MYC* sG4 at 25°C in the presence of *ds*-DNA formed between single-stranded *c-MYC* sG4 and its

complementary oligonucleotide sC4 (10 µM, i.e. 6.6 eq.) and an additional excess of the single-stranded sC4 sequence (10 µM, i.e. 6.6 eq.). **D)** Competitive PAGE studies using **2a** (no DNA samples, lane 1), *c-MYC* Pu22 (20 µM, lanes 2, 5, and 8), TBA (100 µM, lane 3), GC-rich *ds*-DNA (100 µM, lane 6), and self-complementary *ds*-DNA (100 µM, lane 9) with their associated binary mixtures (lanes 4, 7, and 10). The gel was visualised using an excitation wavelength of 457 nm.

Cells stained with 2a and treated with RNase

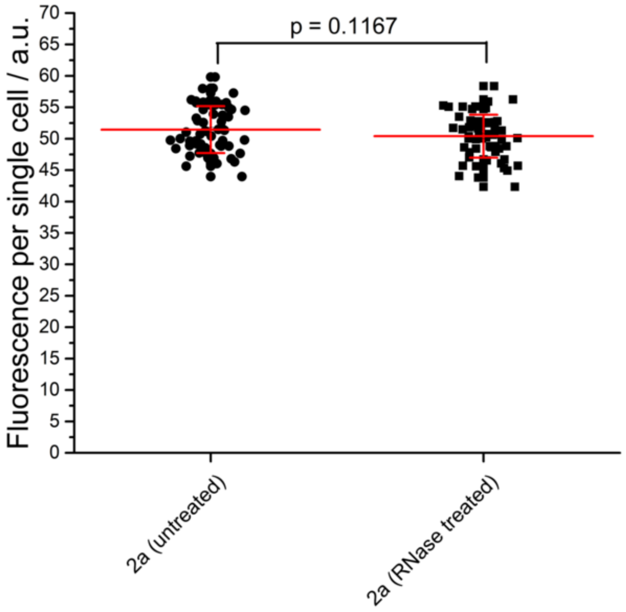

Figure S22. Quantification of fluorescence signal per single cell. Data represent populations of individual cells for each condition of the final experiment: untreated (N = 60 cells) and RNase treated (N = 60 cells). Means ± SD are indicated. Analysis of the data was performed using two-sample t tests and p values are indicated.

Cells stained with 2a and treated with BRACO-19

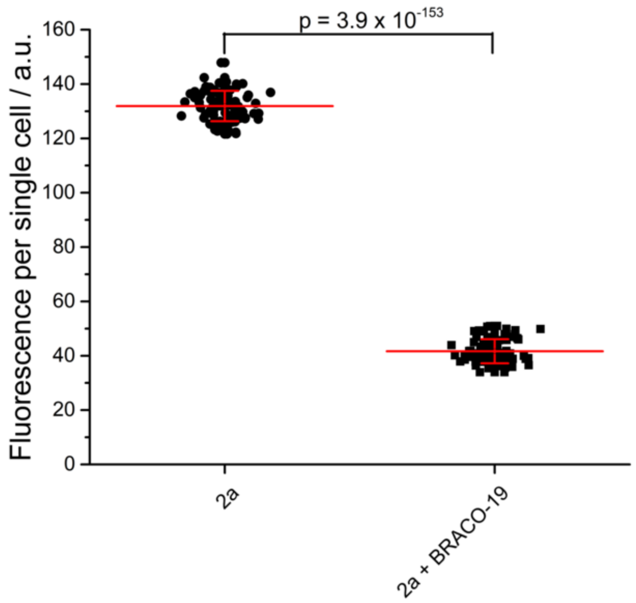

Figure S23. Quantification of fluorescence signal per single cell. Data represent populations of individual cells for each condition of the final experiment: untreated (N = 80 cells) and BRACO-19 (20 µM) treated (N = 80 cells). Means ± SD are indicated. Analysis of the data was performed using two-sample t tests and p values are indicated.

Fibre analysis

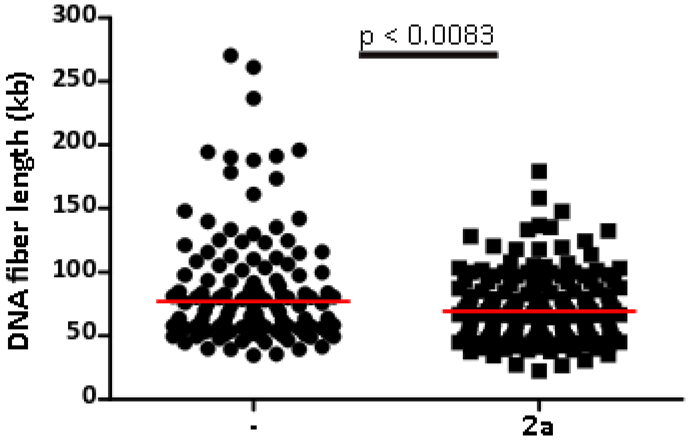

Figure S24. DNA fibre analysis to examine replication speed in mock and **2a**-treated HeLa cells.

2a in aqueous solution at different temperatures

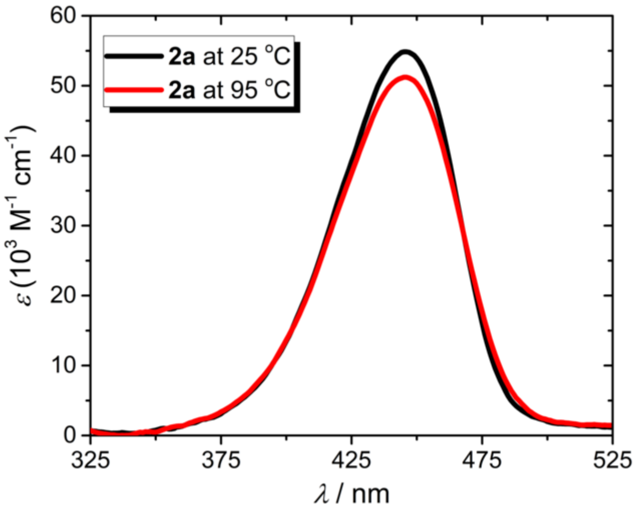

Figure S25. UV/Vis absorption spectra of **2a** at 25 (black line) and 95 °C (red line). $c_{\mathbf{2a}}$ = 3.0 µM, KCl = 100 mM, Tris buffer c = 50 mM, pH = 7.5.

References

[1] Del Villar-Guerra, R., Trent, J. O., and Chaires, J. B. (2018) G-Quadruplex Secondary Structure Obtained from Circular Dichroism Spectroscopy, *Angew Chem Int Ed Engl 57*, 7171-7175.

[2] Mergny, J. L., Li, J., Lacroix, L., Amrane, S., and Chaires, J. B. (2005) Thermal difference spectra: a specific signature for nucleic acid structures, *Nucleic Acids Res 33*, e138.

[3] Sasaki, S., Drummen, G. P. C., and Konishi, G. (2016) Recent advances in twisted intramolecular charge transfer (TICT) fluorescence and related phenomena in materials chemistry, *4*, 2731-2743.

[4] Samanta, S., Halder, S., and Das, G. (2018) Twisted-Intramolecular-Charge-Transfer-Based Turn-On Fluorogenic Nanoprobe for Real-Time Detection of Serum Albumin in Physiological Conditions, *Anal Chem 90*, 7561-7568.
